# Identification, Distribution, and Habitat Suitability Models of Ixodid Tick Species in Cattle in Eastern Bhutan

**DOI:** 10.3390/tropicalmed6010027

**Published:** 2021-02-19

**Authors:** Jamyang Namgyal, Tim J. Lysyk, Isabelle Couloigner, Sylvia Checkley, Ratna B. Gurung, Tenzin Tenzin, Sithar Dorjee, Susan C. Cork

**Affiliations:** 1 District Veterinary Hospital, Department of Livestock, Ministry of Agriculture and Forests, Trashigang 42001, Bhutan; 2 Department of Ecosystem and Public Health, Faculty of Veterinary Medicine, University of Calgary, Calgary, AB T2N 1N4, Canada; timlysyk@gmail.com (T.J.L.); icouloig@ucalgary.ca (I.C.); slcheckl@ucalgary.ca (S.C.); sccork@ucalgary.ca (S.C.C.); 3 Department of Geography, University of Calgary, Calgary, AB T2N 1N4, Canada; 4 National Centre for Animal Health, Department of Livestock, Ministry of Agriculture and Forests, Thimphu 11001, Bhutan; rgur1038@uni.sydney.edu.au (R.B.G.); tenzinvp@gmail.com (T.T.); 5 Khesar Gyalpo University of Medical Sciences of Bhutan, Thimphu 11001, Bhutan; s.dorjee@yahoo.co.nz

**Keywords:** tick distribution, habitat suitability, MaxEnt model, Bhutan

## Abstract

Tick infestation is the most reported parasitological problem in cattle in Bhutan. In May and June 2019, we collected ticks from 240 cattle in two districts of Eastern Bhutan. Tick presence, diversity, and infestation prevalence were examined by morphological identification of 3600 live adult ticks. The relationships between cattle, geographic factors, and infestation prevalence were assessed using logistic regression analyses. Habitat suitability for the tick species identified was determined using MaxEnt. Four genera and six species of ticks were found. These were *Rhipicephalus microplus* (Canestrini) (70.2% (95% confidence interval (CI): 68.7–71.7)), *Rhipicephalus haemaphysaloides* Supino (18.8% (95% CI: 17.5–20.1)), *Haemaphysalis bispinosa* Neumann (8.2% (95% CI: 7.3–9.1)), *Haemaphysalis spinigera* Neumann (2.5% (95% CI: 2–3)), *Amblyomma testudinarium* Koch (0.19% (95% CI: 0.07–0.4)), and a single unidentified *Ixodes* sp. Logistic regression indicated that the variables associated with infestation were: longitude and cattle age for *R. microplus*; latitude for *R. haemaphysaloides*; and altitude and cattle breed for *H. bispinosa* and *H. spinigera*. MaxEnt models showed land cover to be an important predictor for the occurrence of all tick species examined. These findings provide information that can be used to initiate and plan enhanced tick surveillance and subsequent prevention and control programs for ticks and tick-borne diseases in cattle in Bhutan.

## 1. Introduction

Bhutan is a small kingdom located in the Eastern Himalayas between latitudes 26°45′ N and 28°10′ N, and longitudes 88°45′ E and 92°10′ E. It shares borders with China to the north, the Indian states of Assam and West Bengal to the south, Arunachal Pradesh to the east, and Sikkim to the west. It has a total land area of 38,394 km^2^, out of which 70.8% is covered by forest mainly dominated by broadleaf and mixed conifers [1]. It is divided administratively into 20 districts (dzongkhags) and 205 subdistricts (gewogs). The population is 735,553 (as of 30 May 2017), of which 62.2% lives in rural areas, and their livelihoods depend on agriculture and livestock farming [2]. Geographically, the country is characterized by high mountains, dense forests, and fast-flowing rivers that form narrow valleys before flowing out onto the vast north Indian plains. There is a pronounced south–north elevation gradient (100–7500 m above sea level (masl)) and an inverse north–south precipitation gradient (500 to >2000 mm) [1,3]. This extreme variation in altitude and the impact of the North Indian monsoon have resulted in extremely diverse climatic conditions and ecosystems across the country, resulting in six agroecological zones (wet subtropical, humid subtropical, dry subtropical, warm temperate, cool temperate, and alpine) [4]. Vegetation coverage, cropping system, land use, and livestock farming are mainly determined by these agroecological zones.

Livestock farming plays an important role in the rural economy of Bhutan. It not only provides the major income for 49.1% of the population who are subsistence farmers [2] but also contributes toward meeting the increasing demands for dairy products in the country. Cattle, including yaks (*Bos grunniens* L.) and mithuns (*Bos frontalis* Lambert), are the predominant livestock farmed with the population of 0.35 million [5]. They are the most important livestock farmed with their diverse role in providing milk, draught power, transport, and organic manure [6]. The crossbreeds of local indigenous Siri cattle (*Bos taurus indicus* L.) and mithun comprise 56% of the total cattle population while European breeds (*Bos taurus taurus* L.) such as Jersey, Brown Swiss, and Holstein Friesian form 30%. The yaks and their crossbreeds comprise 14% of the country’s cattle population [5].

Tick infestation is a significant challenge for the livestock sector in Bhutan due to direct and indirect impacts on cattle health and production [7]. In 2019, 89% of the parasite related cases reported in cattle were due to tick infestation, and 42% of the cattle in the country were reported to have been treated for it, costing the government approximately 3.18 million Bhutanese Ngultrum (1 USD = Nu.70) for purchasing acaricides alone [8]. Tick-borne diseases like anaplasmosis, babesiosis, and theileriosis are also present in cattle in Bhutan, especially in the southern subtropical districts [7,8]. Despite ticks being prevalent throughout the country, there is currently limited data available on tick diversity, infestation prevalence, tick ecology, and geographic distribution. Current knowledge consists of one unpublished study conducted over a period of one year in cattle in Eastern Bhutan (Dr. Susan C. Cork, personal communication, 2018) and one government report [9] from Western Bhutan. The unpublished study may be the first to identify some tick species present on cattle in Bhutan. They reported *Rhipicephalus microplus* (Canestrini) as the most predominant tick species with *Haemaphysalis* spp. and *Hyalomma* spp. identified to the genus level. The other study by the Regional Livestock Development Center [9] in Western Bhutan identified the genera *Rhipicephalus* (*Boophilus*) spp., *Rhipicephalus* spp., *Haemaphysalis* spp., *Ixodes* spp., and *Amblyomma* spp. but no information on species is available.

In this study, we used targeted field sampling to: (1) determine the presence, diversity, and infestation prevalence of tick species in cattle in two districts of Eastern Bhutan; (2) examine the variation in infestation prevalence of tick species between agroecological zones in Trashigang and Pema Gatshel districts; (3) assess the relationships between cattle, geographic factors, and infestation prevalence using logistic regression analyses; and (4) develop habitat suitability models for tick species identified in Eastern Bhutan, under current environmental conditions, using MaxEnt [10]. The findings from this study are expected to provide information necessary to initiate and plan a targeted tick control program and to develop enhanced surveillance for ticks and tick-borne diseases in cattle in Bhutan.

## 2. Materials and Methods

### 2.1. Study Areas

**Figure 1:**
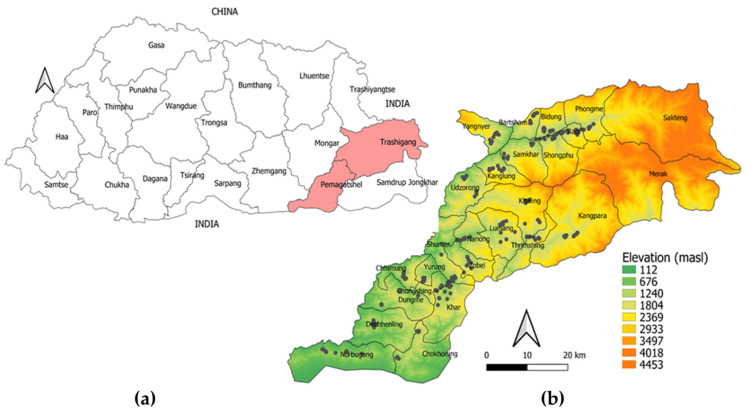
(**a**) Map of Bhutan showing the study area (pink shade) and; (**b**) Elevation map of the study area showing tick sampling sites (black dots). The map was prepared using Quantum GIS, QGIS Development Team (2019), QGIS Geographic Information System, Open-Source Geospatial Foundation Project (http://qgis.osgeo.org (accessed on 28 January 2021)) and was not taken from another source.

The study was conducted in the districts of Trashigang and Pema Gatshel in Eastern Bhutan (Figure 1), covering an area spanning latitudes 26°75′–27°5′ N, and longitudes 91°–91° 85′ E. The study area covered the entire range of agroecological zones represented in Bhutan. These two districts also contain diverse breeds of cattle ranging from the indigenous breed Jaba (*Bos taurus indicus*) common in the lower subtropical plains to the yaks found at the higher alpine areas. The elevation of the study area ranged from 46 to 4571 masl. Trashigang, located toward the north, is predominantly a temperate district characterized by warm summers and cold winters, and shares a border with the Indian state of Arunachal Pradesh in the east. Pema Gatshel, located at the warmer south, is mainly a subtropical district characterized by hot-humid summers and cool winters, and shares a border with the Indian state of Assam in the south. The geographic and climatic features along with the cattle populations of the two districts are summarized in Table 1. Most of the land in both districts is associated with high mountainous terrains separated by narrow valleys. Human settlements and farming activities are generally limited to these narrow valleys and the gentle slopes on the mountains.

### 2.2. Sample Size

The number of sites sampled was determined by the administrative units, i.e., subdistricts/gewogs. Trashigang and Pema Gatshel have 15 and 11 subdistricts, respectively. All subdistricts in the two districts were included for sampling except the two subdistricts in the alpine zone of Trashigang district where we were unable to obtain tick specimens during the field survey conducted in May and June 2019. As the primary objective of the study was the detection of tick species presence, the sample size for each subdistrict was calculated using the formula $n=\left[1-{\left(1-p_{l}\right)}^{\frac{1}{d}}\right]\left(N-\frac{d}{2}\right)+1$ [11], where *n* = required sample size, *N* = number of cattle in subdistricts, *d* = minimum number of tick infested cattle expected in the population, and *p_l_* = probability (0.95) of finding at least one infested cattle in the sample. The number of cattle (*N*) for each subdistrict was obtained from the annual livestock census data [5]. The expected proportion (d) was calculated as 0.3 for all subdistricts based on the tick infestation treatment records from the veterinary information system and the livestock population data of each subdistrict maintained by the Department of Livestock in Bhutan [5]. The sample size calculated for each subdistrict was 10 animals. Since the study area had 24 subdistricts to be sampled, the overall sample size was 240 animals.

### 2.3. Tick Sampling and Specimen Identification

For selecting the required number of animals, the list of households owning cattle in each subdistrict was obtained from subdistrict livestock offices and used as the sampling frame. Ten households owning cattle were selected from each subdistrict using simple random sampling in MS Excel 2016 (Microsoft Excel 2016, Redmond, WA, USA). Overall, this study covered 240 households and collected ticks from 240 cattle. Tick collection was conducted once per household in May–June 2019 in all 24 subdistricts. Prior to tick collection, the objectives of the study were explained to the owners, and verbal consent was obtained to collect ticks from their cattle. All selected households agreed to the sampling. In each farm linked to the selected household, one infested animal that could be properly restrained was selected for sampling (convenience sampling), and 15 ticks were randomly collected from different predilection sites. The ticks collected from each animal were placed separately in Eppendorf tubes containing 70% ethanol and labeled with a unique sample ID that included district and subdistrict codes and the animal number. Other associated information such as age, sex and breed of the animal, GPS coordinates of the location, site altitude, owners’ names, and dates of collection were recorded in an excel spreadsheet for data analyses and future reference. The samples were then transported to the veterinary laboratory at the National Centre for Animal Health (NCAH), Thimphu, Bhutan for morphological identification.

Ticks were identified under a stereo microscope using a two-step process of species identification. Ticks were first identified to genus using the keys described in Matthysee and Colbo [12] and Walker et al. [13]. Ticks were then identified to species using Anastos [14] and Robinson et al. [15] for the members of the genus *Amblyomma*; Matthysee and Colbo [12] and Estrada-Pena et al. [16] for the members of the genus *Rhipicephalus (Boophilus)*; Walker et al. [13] and Anastos [14] for the remaining *Rhipicephalus*; Geevarghese and Mishra [17] and Nutall et al. [18] for the members of the genus *Haemaphysalis*; and Guo et al. [19] for the members of the genus *Ixodes*. The tick specimens, including the voucher specimens, are preserved at the veterinary laboratory, NCAH, Thimphu, Bhutan. Macro photography was done on some of the selected voucher specimens to get representative pictures of the identified ticks.

### 2.4. Environmental Data

Bioclimatic variables were downloaded from the WorldClim database (Version 2, Food and Agriculture Organization, Quebec City, Quebec, Canada) (https://www.worldclim.org/ (accessed on 28 January 2021)) at 30 s (1 km^2^) spatial resolution [20]. The digital elevation–shuttle radar topography information (DEM_SRTM) at 1 arc-second global was downloaded from the United States Geological Survey (USGS) database (https://www.usgs.gov/ (accessed on 28 January 2021)). The most recent land use and land cover data (LULC 2016) for Bhutan at 30 min spatial resolution was obtained from the National Land Commission (NLC) of Bhutan (https://www.nlcs.gov.bt/ (accessed on 28 January 2021)). LULC 2016 uses 17 classes: alpine scrubs (1); broadleaf (2); built-up (3); chuzhing (4); chirpine (5); fir (6); kamzhing (7); lake (8); landslides (9); meadows (10); mixed conifer (11); non built-up (12); orchards (13); rivers (14); rocky outcrops (15); shrubs (16); and snow and glaciers (17). Chuzhing refers to irrigated and bench terraced agriculture land for paddy cultivation, and kamzhing represents cultivated rain-fed dry land primarily used for cereal cropping [21]. DEM_SRTM and LULC 2016 were organized as raster (grid) type files to the spatial extent and resolution of the WorldClim layers. Data preparation was done using raster [22], rgeos [23], and rgdal [24] packages in R (R Core Team, Vienna, Austria) [25]. 

Seven bioclimatic variables (i.e., Bio 2, Bio 3, Bio 4, Bio 7, Bio 13, Bio 14, and Bio 15) were excluded, as they were not deemed ecologically relevant to the study area. Bio 2 (annual mean diurnal range) was excluded due to limited data range (2.3 °C) in the study area. Bio 3 (isothermality) was excluded, as there is little day to night temperature oscillation in the study area. Bio 4 (temperature seasonality) and Bio 7 (annual temperature range) was excluded, as their information is included in other temperature variables. Bio 13 (precipitation of the wettest month), Bio 14 (precipitation of the driest month), and Bio 15 (monthly precipitation variation) were excluded, as the precipitation in Bhutan is seasonal depending on the North Indian monsoon. The seasonal variations are more important than the monthly variations; therefore, the precipitation of the quarters was used. The environmental variables used in the MaxEnt are listed in Table 2.

### 2.5. Statistical Analyses

The raw data were collated in Excel spreadsheets and imported into R (R Core Team, Vienna, Austria) [25] for analyses. The tick species considered for statistical analyses were *R. microplus*, *R. haemaphysaloides*, *H. bispinosa*, and *H. spinigera*. The remaining two species, *A. testudinarium,* and *Ixodes* sp. were excluded from analyses, as they were infrequently collected. Descriptive analysis was performed with the entire dataset to calculate proportions and frequencies. Tick infestation prevalence was calculated as the number of cattle infested with ticks divided by the total number of cattle examined among the households sampled [26]. The 95% binomial confidence interval for the infestation prevalence was calculated using the Clopper–Pearson exact method using the “PropCI” package [27]. Pearson’s chi-squared test using the native “stats” package [25] was performed to compare the difference in proportions of tick infestation prevalence between the districts.

Logistic regression analyses were conducted using the cattle age (categorized as young or adult), sex, breed (categorized as European or indigenous), site altitude (in 100 m), latitude (in 1/10 decimal degrees), and longitude (in 1/10 decimal degrees) as the explanatory variables against the binary outcome variables of infestation prevalence of each tick species (categorized as infested or not). Correlation among the explanatory variables was assessed using the “Hmisc” package [28]. The explanatory variables with *p*-value ≤ 0.25 in univariable analyses were selected for multiple logistic regression analysis. A forward stepwise approach was used to manually build the final multiple logistic regression models. First, a variable with the smallest *p*-value in the univariable analysis was included into the model. Then, each of the remaining variables was individually added to the model to assess whether its addition improved the fit of the model significantly at *p*-value ≤ 0.05. A likelihood ratio test was used to select the variable that had the greatest improvement in the likelihood ratio statistic, and the process was repeated. Only the variables at the *p*-value ≤ 0.05 level of significance were retained in the final model. Multicollinearity of the explanatory variables in the models was assessed using the variance inflation factor (VIF) at the cut-off of 2.5 [29]. Interactions were assessed by adding a cross-product term (i.e., Latitude × Longitude). The odds ratio (OR) and its 95% confidence interval (CI) of the variables associated with the outcome variables were calculated from the final models. The goodness-of-fit for the final models were evaluated using the “LogisticDx” package [30]. The residual analysis of the final models was done using the “car” package [31].

### 2.6. Habitat Suitability Modeling

The habitat suitability modeling was conducted using MaxEnt v3.4.1 (New York, NY, USA) [10] in R (R Core Team, Vienna, Austria) [25] using the “dismo” package [32] and the “prepPara” function [33]. The presence points (i.e., latitude and longitude pair) for four tick species, *R. microplus* (204 points), *R. haemaphysaloides* (91 points), *H. bispinosa* (72 points), and *H. spinigera* (28 points) were used for the modeling. The presence data were transformed into the spatial data frame using the coordinates function in the “sp” package [34]. All environmental layers were stacked as raster layers and restricted to the study area using crop and mask functions in the “raster” package [22]. 

In the first step, MaxEnt was run with iterations and background points set to default using all the environmental variables that were transformed into the hinge feature. Variables contributing less than 1% to the increasing training gain or less than 1% permutation importance were considered non-significant and excluded from further analyses [35]. Variables selected from the first MaxEnt run for each tick species were examined for correlation using the corr.test function in the “psych” package [36].

In the second step, for each tick species, competing models were built using all selected variables and then subsequently reducing the model by removing the less important variables based on Akaike information criteria (AIC) [37]. AIC was calculated using the cal.aicc function from the “ENMeval” package [38], and model evaluations were done using the evaluate function from the “dismo” package [32]. Competing models for each tick species were then compared using threshold independent receiver operating characteristics (ROC) graphs [39], correlation, and the AIC [37,40]. The best model for each tick species were then selected based on AIC values, and whenever AIC values were indecisive, the most parsimonious models were selected.

Using “rasterVis” [41] and “RColorBrewer” [42] packages, the habitat suitability of the best model for each tick species was reclassified and mapped following Zuliani et al. [35] into 5 classes: class 1 (very low) for the probability level (p) between 0 and 0.2; class 2 (low) for p between 0.2 and 0.4; class 3 (moderate) for p between 0.4 and 0.6; class 4 (high) for p between 0.6 and 0.8; and class 5 (very high) for p between 0.8 and 1.0. For the best models, the response curves of the predictors were qualitatively assessed to understand the relationship between the variation in individual variables and the probability of tick species occurrence. 

## 3. Results

### 3.1. Tick Diversity and Infestation Prevalence

**Figure 2:**
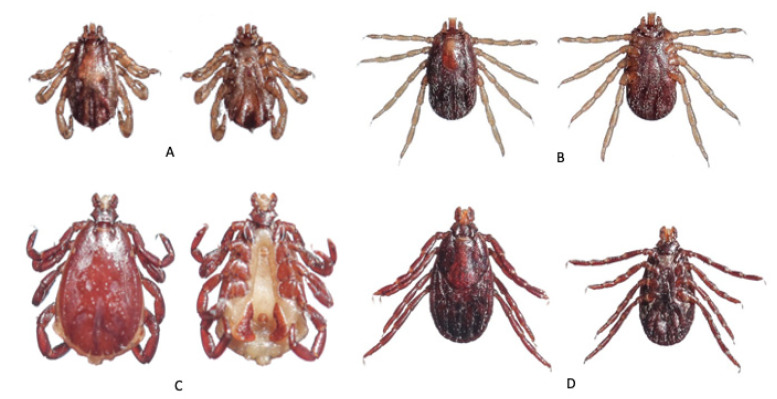
Dorsal and ventral views of male (**A**) and female (**B**) *Rhipicephalus microplus*; and male (**C**) and female (**D**) *Rhipicephalus haemaphysaloides*.

**Figure 3:**
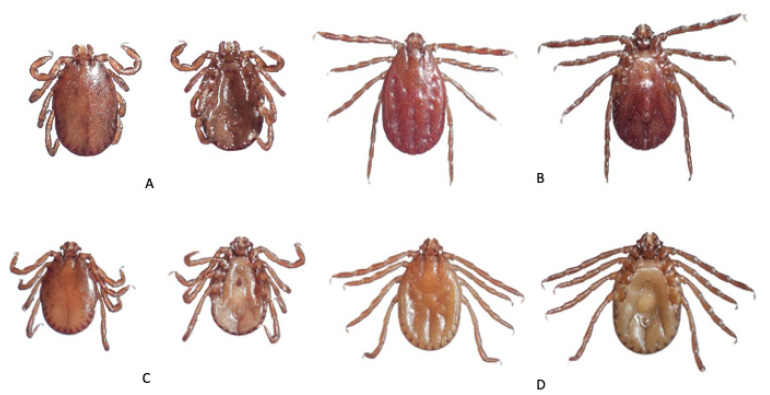
Dorsal and ventral views of male (**A**) and female (**B**) *Haemaphysalis bispinosa*; and male (**C**) and female (**D**) *Haemaphysalis spinigera*.

**Figure 4:**
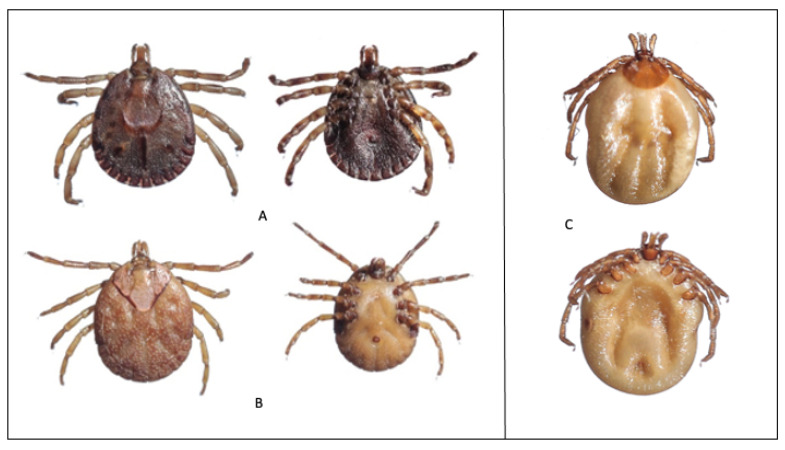
Dorsal and ventral views of male (**A**) and female (**B**) of *Amblyomma testudinarium*; and female (**C**) *Ixodes* sp.

A total of 3600 ticks were collected and identified to four genera and five species (Table 3 and Figure 2, Figure 3 and Figure 4). The majority were *Rhipicephalus microplus* (Canestrini) (70.3%) followed by *Rhipicephalus haemaphysaloides* Supino (18.8%), *Haemaphysalis bispinosa* Neumann (8.2%), and *Haemaphysalis spinigera* Neumann (2.5%). The remainder were seven *Amblyomma testudinarium* Koch and one unidentified species of *Ixodes* sp. *Amblyomma testudinarium* and *Ixodes* sp. were found only in Pema Gatshel and Trashigang district, respectively. The variation in proportions of tick infestation prevalence between the two districts is given in Table 4.

### 3.2. Factors Associated with Tick Infestation in Cattle 

The characteristics of the cattle sampled and geographic variables are shown in Table 5. For *R. microplus*, simple logistic regression (Table S1) indicated that infestation varied between cattle age groups, but not between sexes or breeds. The infestation declined with each of increasing altitude, latitude, and longitude in simple logistic regression (Table S1). The odds of cattle being infested with *R. microplus* were 5.6 times more likely in the young animals than the adult animals (OR = 5.6 (95%CI: 1.5–35.5)), and there was a 40% (OR = 0.6 (95%CI: 0.42–0.75)) lower likelihood of *R. microplus* infestation with every 1/10th degree increase in longitude, when the other variable in the model was held constant, respectively (Table 6).

For *R. haemaphysaloides*, simple logistic regression indicated that infestation varied among cattle age groups, but neither sex nor breed (Table S1). It also indicated that infestation increased with each of altitude, latitude, and longitude (Table S1). However, the final multiple logistic regression showed that the odds of cattle being infested with *R. haemaphysaloides* increased 2.0 times with every 1/10th degree increase in latitude (OR = 2.0 (95%CI: 1.7–2.5)) (Table 6).

For both *Haemaphysalis* species (i.e., *H. bispinosa* and *H. spinigera*), simple logistic regression indicated no significant effect of cattle age and sex on infestation, but a significant effect of the breed (Table S1). Both species showed no relationship with latitude or longitude, but a significant effect of altitude (Table S1). However, *H. bispinosa* indicated a significant positive relationship with altitude while *H. spinigera* indicated a significant negative relationship with altitude (Table S1). In the multiple logistic regression analysis, infestation of both *Haemaphysalis* species varied with both altitude and cattle breed. The odds of cattle being infested with *H. bispinosa* were 1.8 times more likely in the indigenous breeds than that of the European breeds (OR = 1.8 (95%CI: 1.0–3.3)), and the infestation increased 1.1 times with every 100 m increase in altitude (OR = 1.1 (95%CI: 1.0–1.1)) when the other variable in the model was held constant, respectively (Table 6). For *H. spinigera*, the odds of cattle being infested were 2.7 times more in the indigenous breeds than that of the European breeds (OR = 2.7 (95%CI: 1.2–6.4)), and there was 20% (OR = 0.8 (95%CI: 0.7–0.9)) lower likelihood of *H. spinigera* infestation with every 100 m increase in altitude, when the other variable in the model was held constant, respectively (Table 6).

### 3.3. Habitat Suitability Modeling

**Figure 5:**
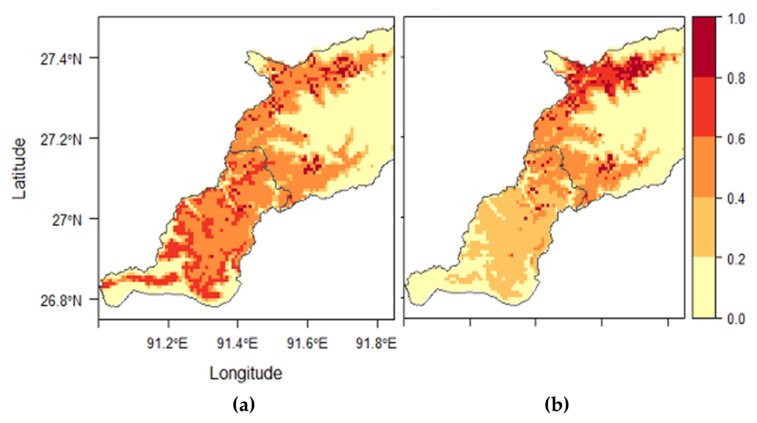
Habitat suitability maps developed by the best models (**a**) for *Rhipicephalus microplus* using land use and land cover (LULC) and digital elevation–shuttle radar topography information (DEM_SRTM) and (**b**) for *Rhipicephalus haemaphysaloides* using LULC, Bio 16, and Bio 10.

For *R. microplus*, three variables (i.e., DEM_SRTM, LULC, and Bio 18) achieved more than 1% contribution and permutation importance in the first MaxEnt run (Table S2). There was a high correlation (r_s_ = −0.93) between DEM_SRTM and Bio 18 (Table S3). Four competing models were built with the selected variables (Table S4). The best model showed that elevation was the most important predictor at 56.1%, followed by land cover at 43.9% (Table 7). The analysis of the response curve showed that the probability of *R. microplus* occurrence increased with elevation between 500 and 1000 masl, then declined (Figure S1). As with land cover, the probability of occurrence was most significant in land cover classes classified as kamzhing (0.84) and meadows (0.81) (Figure S1). Shrubs were associated with a 0.55 probability of occurrence. Overall, 49.7% of the study area was predicted as moderate to high suitable areas for *R. microplus* (Table S5). Based on the habitat suitability map (Figure 5), the northeastern part of the study area with elevation above 2000 masl and the southernmost part with elevation less than 500 masl were predicted as very low suitable areas for *R. microplus* occurrence.

For *R. haemaphysaloides*, six variables (i.e., LULC, Bio 18, Bio 16, Bio 10, DEM_SRTM, and Bio 8) achieved more than 1% contribution and permutation importance in the first MaxEnt run (Table S2). Correlation analyses indicated that all variables were highly correlated (r_s_ > 0.90) (Table S3). Eighteen competing models were built using the selected variables (Table S4). The best model showed that land cover was the most important predictor at 46%, followed by Bio 16 (precipitation of the wettest quarter) and Bio 10 (temperature of the warmest quarter) at 43.1% and 10.9%, respectively (Table 7). The analysis of the response curve showed that the probability of occurrence was most significant in land cover classes classified as kamzhing (0.82), followed by shrubs (0.6) and chuzhing (0.59) (Figure S2). The probability of occurrence increased with the temperature of the warmest quarter (Bio 10) between 16 and 25 °C, beyond which there was a decline (Figure S2). The probability of occurrence also increased with precipitation of the wettest quarter (Bio 16) between 400 and 1100 mm, then declined when Bio 16 exceeded 1200 mm (Figure S2). Overall, 27.4% of the study area was predicted as moderate to high suitable areas for *R. haemaphysaloides* (Table S5). Based on the habitat suitability map (Figure 5), the northeastern and the southernmost part of the study area were predicted as low suitable areas for *R. haemaphysaloides* occurrence.

**Figure 6:**
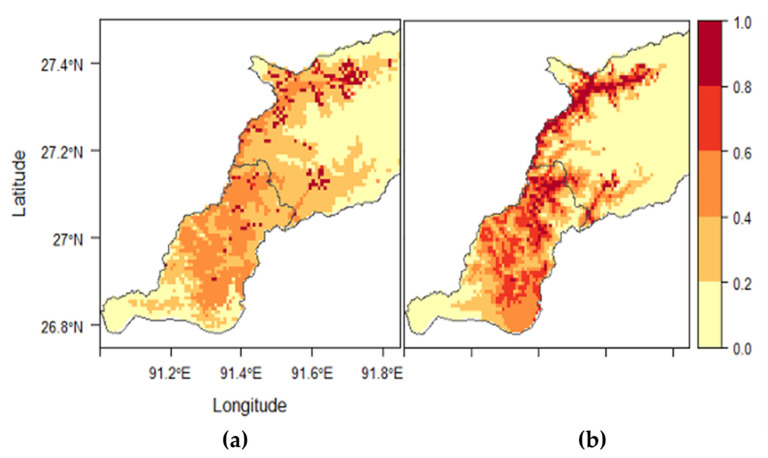
Habitat suitability maps developed by the best models (**a**) for *Haemaphysalis bispinosa* using LULC, Bio 18, and Bio 16 and (**b**) for *Haemaphysalis spinigera* using LULC, Bio 19, and Bio 16.

For *H. bispinosa*, five variables (i.e., LULC, Bio 18, Bio 16, Bio 12, and Bio 11) achieved more than 1% contribution and permutation importance in the first MaxEnt run (Table S2). Correlation analyses indicated that all variables were highly correlated (r_s_ > 0.90) (Table S3). Thirteen competing models were built using the selected variables (Table S4). The best model showed that land cover was the most important predictor at 63.2%, followed by Bio 18 (precipitation of the warmest quarter) and Bio 16 (precipitation of the wettest quarter) at 30.6% and 6.2%, respectively (Table 7). The response curve showed that the probability of occurrence was most significant in Kamzhing (0.83) and meadows (0.82) (Figure S3). Shrubs and Chuzhing had 0.64 and 0.55 probabilities, respectively. The probability of occurrence increased with precipitation of the warmest quarter (Bio 18) between 600 and 2000 mm, beyond which there was a decrease (Figure S3). The probability of occurrence also increased with precipitation of the wettest quarter (Bio 16) between 400 and 900 mm, then declined when Bio 16 exceeded 1600 mm (Figure S3). Overall, 24.9% of the study area was predicted as moderate to high suitable areas for *H. bispinosa* (Table S5). Based on the habitat suitability map (Figure 6), the northeastern and the southernmost part of the study area were predicted as low suitable areas for *H. bispinosa* occurrence.

For *H. spinigera*, seven variables (i.e., LULC, Bio 8, Bio 11, Bio 12, Bio 16, Bio 17, and Bio 19) achieved more than 1% contribution and permutation importance in the first MaxEnt run (Table S2). All variables were highly correlated (r_s_ > 0.70) (Table S3). Twenty-six competing models were built using the selected variables (Table S4). The best model showed that Bio 16 (precipitation of the wettest quarter) was the most important variable at 45.7%, followed by Bio 19 (precipitation of the coldest quarter) and land cover at 33.4% and 20.4%, respectively (Table 7). The response curve showed that the probability of occurrence increased with precipitation of the wettest quarter (Bio 16) at 400 mm, then declined when Bio 16 exceeded 1600 mm (Figure S4). With the precipitation of the coldest quarter (Bio 19), the probability of occurrence increased between 15 and 40 mm, beyond which there was a decrease (Figure S4). As with land cover classes, the probability of occurrence was 0.88 in kamzhing and 0.67 with shrubs (Figure S4). Overall, 33.2% of the study area was predicted as moderate to high suitable areas for *H. spinigera* (Table S5). Based on the habitat suitability map (Figure 6), the northeastern and the southernmost part of the study area were predicted as low suitable areas for *H. spinigera* occurrence.

## 4. Discussion

The level of tick infestation in animals is generally influenced by both host and environmental factors. The host (cattle in our study) factors such as age, sex, and breed can influence the susceptibility of animals to tick infestation [43,44]. Ticks are also generally dependent on the temperature and rainfall for their development and activity [16]. In Bhutan, climatic variables such as temperature and rainfall are primarily determined by altitude and latitude [45]. Thus, in the first part of our study, the relationships between cattle and geographic factors and infestation prevalence were assessed. In the second part, we modeled the relationship between tick species presence and the environmental variables, using the MaxEnt [10] species distribution modeling to identify environmental factors associated with the geographical distribution of the tick species found in the study area. MaxEnt modeling was selected because it can build a reliable model of species distribution using presence-only data and environmental variables without assuming species absence in locations not sampled or surveyed [46].

In this study, *R. microplus* was found to be the most predominant tick species infesting cattle in Eastern Bhutan (Table 3) and was collected from all subdistricts. Our study findings agreed with a previous unpublished study, which found *R. microplus* to be the primary tick species in cattle in the surveyed areas of eastern Bhutan. Further, a tick survey conducted in the western region of Bhutan also found *R. microplus* to be the most prevalent tick species [9]. This tick species is also one of the most predominant tick species infesting livestock in India [47]. Moreover, this tick species is distributed throughout the world, especially in tropical and subtropical regions, and considered to be the most important tick species of cattle in the world [48,49]. Recently, it was recognized that *R. microplus* is a complex species that is comprised of at least five taxa: *R. australis, R. annulatus, R. microplus* clade A, *R. microplus* clade B, and *R. microplus* clade C [50,51]. However, the *R. microplus* we have collected was identified based on morphological keys [12,16], and further studies should be conducted to determine which clade of *R. microplus* it belongs to.

*Rhipicephalus microplus* was found more frequently in animals sampled in Pema Gatshel (98.2%) compared with those sampled in Trashigang (78.3%). Generally, this one-host tick species prefers warm and humid conditions [52], and temperature and rainfall are the most important climatic factors driving its geographic distribution [53,54]. Pema Gatshel, located at the warmer south, has the majority of its area dominated by subtropical climate with higher temperatures, abundant rainfall, and high relative humidity, while Trashigang is mainly characterized by a temperate climate with moderate temperature, rainfall, and relative humidity. This difference in the climatic conditions could be one factor that could have led to the greater prevalence of *R. microplus* in Pema Gatshel.

The infestation prevalence of *R. microplus* was found to decrease with increasing longitude (Table 6). Geographically, the longitudes of our study area ranged from 91° to 92° E. The areas between the longitudes 91° and 91.5° E are at an elevation of below 2000 m. Between the longitudes 91.5° and 92° E, the areas are at an elevation of more than 2000 m. This variation in elevation toward the east with increasing longitude could be the reason for the decrease in *R. microplus* infestation as *R. microplus* is known to avoid higher altitudes such as mountains and plateaus, where low temperatures are prevalent [55,56]. Elevation was also found as the most important environmental variable in the best habitat suitability model generated for *R. microplus*. The highest probability of its occurrence corresponded to the low elevation range (500–1000 masl) and decreased after that threshold. This elevation range corresponds to the humid subtropical zone (600–1200 masl) in Bhutan, characterized by an annual mean temperature of 19.5 °C and an annual rainfall of 1200–2500 mm [1]. This agrees with a study from Zimbabwe, where elevation was found as the most influential factor for the geographic distribution of *R. microplus* and *R. decoloratus* [54]. In Bhutan, areas with higher elevation are characterized by colder temperatures and low rainfalls, and that might result in such areas to be less suitable habitats for *R. microplus*.

A greater prevalence of *R. microplus* was observed in younger cattle compared with that of adult cattle (Table 6), which is consistent with the similar studies conducted in the neighboring Indian state of West Bengal [57], Bangladesh [58], and Nigeria [59]. However, studies conducted in Pakistan [60], Ethiopia [61], Egypt [43], and Nigeria [62] indicated lower prevalence in young cattle due to factors such as frequent grooming by dams and innate immunity. In our study area, those calves that are reared in the stall-fed systems are deprived of grooming as the farmers tether them at a safe distance from their dam to avoid milk suckling. Furthermore, young calves, especially the males, are the least attended by farmers, unlike the milking adult females. In most cattle owning households in the study area, calves and young heifers are often let out for grazing in nearby pastures and forests while milking adults are kept tethered around the homestead. Lack of maternal grooming and free access to pastures and forests likely increase the risk of tick infestation, and this could be the reason for the greater prevalence of *R. microplus* in younger animals in this study.

*Rhipicephalus haemaphysaloides* was the second most predominant tick species in this study (Table 3) and was collected from 20 subdistricts. This typical three-host tick has a limited geographical distribution in the world [63]; however, it is widely distributed in the neighboring Indian states bordering Bhutan [47] and China [64]. Unlike *R. microplus*, the prevalence of *R. haemaphysaloides* was greater in animals sampled from Trashigang (56.9%) compared with those sampled in Pema Gatshel (15.4%). It also showed a positive association with latitude indicating northerly distribution in the study area (Table 6). Trashigang, located toward the north in the study area, is at a higher elevation compared with Pema Gatshel. Temperatures and rainfalls are also comparatively low. Generally, temperature is considered to be the key climatic factor influencing the biological performance of this tick [63]. However, our findings suggest that *R. haemaphysaloides* might be tolerating cooler temperatures than other tick species at some point in their life cycle. In the habitat suitability modeling, *R. haemaphysaloides* presence was related to Bio 10 (temperature of the warmest quarter) and Bio 16 (precipitation of the wettest quarter) with the highest probability of occurrence at 25 °C and 1100 mm precipitation, respectively. Generally, the relative humidity plays a significant role in regulating the questing activity of ticks, and, further, the mortality of ticks also depends on the loss of water [65]. This tick species is an exophilic tick that may lose water while questing for a host, and thus its survival may be dependent on its ability to retain or gain water. Therefore, precipitation might play a critical role in the survival of this tick in the environment as the relative humidity is largely influenced by precipitation.

*Haemaphysalis bispinosa* was the third most predominant tick species identified in this study (Table 3) and was collected from 22 subdistricts. In South Asia, this tick is distributed in India, Nepal, Sri Lanka, Pakistan, Bangladesh, and Myanmar [66]. In India, it is widely distributed throughout the country and has been reported from the Indian states of Assam, Arunachal Pradesh, West Bengal, and Sikkim [17,47,67] that border Bhutan. In China, this tick has been considered to exist in the southern parts of the country, but most of the *H. bispinosa* reported in the Chinese literature are considered to be, in fact, *H. longicornis* [68]. *Haemaphysalis spinigera* was collected from 14 subdistricts in the study area. This tick is found in the foothills of the Central and Eastern Himalayan region, through Nepal to West Bengal in India [69]. It is widely distributed in India and has been reported from the Indian states of Assam and West Bengal [17,47], which border Bhutan.

The prevalence of both *H. bispinosa* and *H. spinigera* was greater in indigenous breeds compared to European breeds. This could be attributed to the difference in preferred management systems used for European and indigenous breeds. The European breeds are mostly reared in a stall-fed system with limited access to forests, whereas the indigenous breeds are mostly reared in a free-grazing system with easy access to forests. The risk of exposure to ticks becomes greater when cattle graze in the forests. Studies have observed a higher prevalence of tick infestation in grazing cattle [58,70,71]. Furthermore, indigenous breeds of cattle in Bhutan are generally considered resistant to many pests and diseases, and the management practices like grooming, brushing, and acaricide application, are rarely practiced by the farmers [7].

*Haemaphysalis bispinosa* infestation increased with increasing altitude while *H. spinigera* infestation decreased with increasing altitude (Table 6). This might indicate that *H. bispinosa* can tolerate a cooler temperature at some point in its life cycle while *H. spinigera* prefers low altitude subtropical areas. However, altitude is not the only driving factor in the distribution of this tick species. Climatic factors such as temperature, rainfall, relative humidity, vegetation, and host availability also influence its distribution [72]. In Bhutan, the high mountains and narrow valleys cause an extreme variation in climate, and temperature is mainly affected by altitude and precipitation by latitude [45]. Further, the types of vegetation in the country are thought to be determined by the terrain and local climatic conditions; therefore, in the absence of detailed data on these various climatic and environmental factors, it is difficult to interpret what factors largely drive the distribution of ticks in Bhutan. Nevertheless, altitude could be one of the main driving factors determining the habitat suitability of tick species as it is believed to change the temperature by 0.5 °C with every 100 m change in altitude [45]. 

The best habitat suitability model generated for *H. bispinosa* showed that Bio 18 (precipitation of the warmest quarter) and Bio 16 (precipitation of the wettest quarter) were the two variables, besides land cover, predicting its distribution. For *H. spinigera*, Bio 16 (precipitation of the wettest quarter) and Bio 19 (precipitation of the coldest quarter) were the two important variables, besides land cover. This indicates that the distribution of both *Haemaphysalis* species was related to precipitation variables and land cover. The pattern of influence by Bio 16 (precipitation of the wettest quarter) was similar for both species—the probability of occurrence increased at 400 mm and then decreased when Bio 16 exceeded 1600 mm. Other studies from the neighboring countries like Bangladesh and India had similar findings for these two *Haemaphysalis* species. *Haemaphysalis bispinosa* seems to thrive well in areas with high summer rainfall in Bangladesh [73]. In India, the population of the engorged adults increased during the monsoon seasons when there is a lot of rain [17]. Similarly, *H. spinigera* is also known to prefer wet and moist habitats with heavy to moderate rainfall [17]. In South India, where Kysanuar forest disease (KFD) is endemic, the nymphal activity of *H. spinigera* (the principal vector of KFD) peaks in spring and summer seasons coinciding with the onset of monsoons [74]. Further, a habitat suitability modeling study (preprint) [75] from South India for *H. spinigera* has identified precipitation variables such as annual precipitation and wettest month precipitation as the important climatic variables influencing its geographical distribution. These findings indicate that precipitation is one of the important climatic factors that determine the distribution of *H. bispinosa* and *H. spinigera*. 

The habitat suitability models developed indicated that the land cover classes such as kamzhing, meadows, chuzhing, and shrublands were suitable habitats for the tick species modeled. This might be associated with the presence of a host in these types of land. Kamzhing is the most important land type for dry land agricultural farming in Bhutan. Besides cultivating cereal crops, kamzhing is also used for growing fodder crops like oats and clover, especially in winters. Cattle are usually tethered and allowed to graze on the small patches of grasses and weeds in the kamzhing areas, and this might explain why kamzhing is a suitable land type for ticks, especially for a one-host tick like *R. microplus*. The high probability of tick(s) occurrence in the meadows could also be due to host availability. Most of the indigenous cattle breeds reared in a free grazing system are grazed in the meadows, and frequently, wild cervids like deer also graze in the meadows. Chuzhing, at least theoretically, should not be a suitable habitat as ticks would not be able to withstand flooding. However, the entire area categorized as chuzhing is not under rice cultivation. The insufficient irrigation supply, farm labor shortage, erratic rainfall, and human–wildlife conflicts, among many other factors, have pushed many farmers to leave their chuzhing fallow [76]. Consequently, these fallow lands have become a grazing area for domestic animals like cattle and wild cervids like deer. Occasionally, some farmers also grow fodder grasses on chuzhings that could not be utilized for rice cultivation. This changing pattern of land use might be the reason for chuzhing featuring as one of the suitable habitats for ticks. Shrublands were also identified as a suitable land type for some of the tick species modeled. Generally, shrublands in Bhutan are suitable for tick–host interactions as they are normal grazing areas for cattle and wild herbivores such as deer. Further, shrublands provide a conducive environment for small mammals, especially rodents, which can serve as reservoir hosts for the larvae and nymphs of multihost tick species.

For all tick species modeled, the northeastern and the southernmost parts of the study area were predicted to be unsuitable areas. The northeastern region is the area with higher elevations where temperatures and rainfall are low. In this region, the vegetation is predominantly mixed conifer forests. The low temperatures, scanty rainfall, and the occasional snow and frosts might be the limiting factors for tick survival. However, there might be some other species of ticks such as *Hyalomma* spp., which can tolerate high elevations. *Hyalomma* spp. were collected in Eastern Bhutan in areas with high elevation (above 2700 masl) (Dr. Susan C. Cork, personal communication, 2018). Therefore, additional studies using other sampling methods such as flagging and dragging may have to be conducted for at least a year to ascertain whether these areas are unsuitable for ticks. The southernmost part predicted as unsuitable areas are the subtropical floodplains at a very low elevation (less than 500 masl). The vegetation in these areas is predominantly broadleaf forests. Climatically, these areas will be suitable for ticks, but the host unavailability (especially the bovines) might be the limiting factor. There are no human activities such as settlement and farming in these areas. The frequent flooding during the monsoon will also be one of the limiting factors. However, there are some wild animals inhabiting these areas, which might be potential hosts for some species of ticks. Therefore, future efforts must be targeted toward tick collection from wild animals and the environment in these areas to understand the habitat suitability better.

Seven specimens of *A. testudinarium* were collected from seven subdistricts in Pema Gatshel, and one unidentified specimen of *Ixodes* was collected from a subdistrict in Trashigang. *Amblyomma testudinarium* is considered to be a rare tick species reported only from Asian countries like Malaysia, India, Japan, Korea, and China [47,77,78]. In India, it has been collected from cattle, mithuns, yaks, and wild animals such as the tiger, wild boar, barking deer, and elephant in Assam, Arunachal Pradesh, Mizoram, and West Bengal [47,79]. These reports suggest that this tick species is predominantly found on the animals that live in and around the forests of the Himalayan foothills. In this study, *A. testudinarium* was found only in the southeastern district of Pema Gatshel. Since this study lacked year-round sampling and was conducted only in cattle, it is difficult to interpret why *A. testudinarium* was not collected from Trashigang. As with *Ixodes*, there are 11 valid species reported from India [47], 24 valid species from China [19], and 15 valid species from Nepal [80]. This tick can infest a wide range of hosts, including domestic animals and humans, small mammals, wildlife such as deer, birds, and reptiles [80]. However, in this study, only one specimen of *Ixodes* was found on cattle. This could have been biased due to our sampling procedure as tick collection was done only from the cattle in May–June 2019.

This study has some limitations. Since the primary aim of our study was to understand the presence and diversity of tick species in cattle, our sampling was biased toward areas dominated by cattle rearing. The presence data used in this modeling study were based on tick collection from cattle, and it may not represent the exact location from where tick(s) originated. Therefore, future studies should include tick sampling from the environment and other hosts. The sampling for this study was conducted only once in May and June 2019 as this time period is thought to be the season for peak tick infestation in cattle in Bhutan. More species might have been found if the sampling was conducted throughout the year. To reduce these knowledge gaps, future studies should focus on active surveillance for some years targeting a wider range of hosts. Other sampling methods like flagging and dragging on vegetation should also be conducted to improve the knowledge of tick species diversity. The three-host ticks like *R. haemaphysaloides*, *H. bispinosa,* and *H. spinigera* depend on multiple hosts to complete their life-cycle; however, we collected ticks only from cattle, and the status of other potential hosts that these ticks might be feeding on is not known. In Bhutan, tick collection from a wider range of hosts could be done in collaboration with wildlife officials who often encounter wild animals and birds during rescue and relocation operations. The resampled bioclimatic variables may not represent the extreme climatic variations in the mountainous terrain of Bhutan, thereby increasing the uncertainty of the models. Therefore, more local climate or weather data should be used for future modeling studies. Further, the MaxEnt approach, as a correlative species distribution model, estimates the realized niche but not the fundamental niche of the species [81]; therefore, potential habitat is likely to be more than what is predicted by the habitat suitability maps. Nevertheless, MaxEnt is a powerful software tool for habitat suitability modeling with several advantages that include the need for few presence points and the ability to use both continuous and categorical variables [10].

## 5. Conclusions

The habitat suitability models developed in this study identified some potential climatic and environmental factors that can be used to predict the current distribution of common tick species in Eastern Bhutan. Land cover types such as kamzhing, meadows, chuzhing, and shrubland were identified as the preferred habitat of these tick species. Elevation was found to be the most important environmental variable influencing the distribution of *R. microplus*. Bio 10 (temperature of the warmest quarter) and Bio 16 (precipitation of the wettest quarter) were identified as the most important climatic variables driving the distribution of *R. haemaphysaloides*. Precipitation variables (i.e., precipitation of the warmest, the coldest, and the wettest quarters) were identified as the important climatic variables for two *Haemaphysalis* species (i.e., *H. bispinosa* and *H. spinigera*). Further, the habitat suitability maps for the tick species studied can be used for planning a targeted tick surveillance in Eastern Bhutan. Overall, the findings are expected to inform surveillance plans and help in the development and implementation of a targeted tick control program and subsequent prevention and control programs for ticks and tick-borne diseases in cattle in Bhutan. 

## Figures and Tables

**Table 1:** Geographic and climatic characteristics of the study area.

Characteristic	Trashigang	Pema Gatshel	Total
Total land (km^2^)	2204	1023	3227
Agro-ecological zones (in % of land area)			
Wet Subtropical (100–600) *	0.1	20.9	6.7
Humid Subtropical (600–1200)	4.4	41.7	16.2
Dry Subtropical (1200–1800)	14.8	28.1	19
Warm Temperate (1800–2600)	29.2	9.3	22.9
Cool Temperate (2600–3600)	32.7	0	22.3
Alpine (>3600–7500)	18.8	0	12.8
Forest coverage (in %)	87.6	81.6	
Vegetation type	Temperate - broadleaf	Broadleaf	
Climate	Warm and temperate	Hot and humid	
Annual rainfall (in mm)	1115.6	1649.6	
Annual Temperature (min-max in °C)	10.3–24.2	13.1–22.1	
Cattle population	40,685	8252	

* Numbers in bracket are the elevation range in masl.

**Table 2:** Environmental variables used in building MaxEnt habitat suitability models.

Variable, Unit	Code	Source
Annual mean temperature, °C	Bio 1	WorldClim
Maximum temperature of warmest month, °C	Bio 5	WorldClim
Minimum temperature of coldest month, °C	Bio 6	WorldClim
Mean temperature of wettest quarter, °C	Bio 8	WorldClim
Mean temperature of driest quarter, °C	Bio 9	WorldClim
Mean temperature of warmest quarter, °C	Bio 10	WorldClim
Mean temperature of coldest quarter, °C	Bio 11	WorldClim
Annual precipitation, mm	Bio 12	WorldClim
Precipitation of wettest quarter, mm	Bio 16	WorldClim
Precipitation of driest quarter, mm	Bio 17	WorldClim
Precipitation of warmest quarter, mm	Bio 18	WorldClim
Precipitation of coldest quarter, mm	Bio 19	WorldClim
Elevation (in masl)	DEM_SRTM	USGS
Land Use and Land Cover 2016 *	LULC 2016	NLC, Bhutan

* Categorical variable.

**Table 3:** Tick species sampled from farm animals in Trashigang and Pema Gatshel districts, Eastern Bhutan (2019).

Tick Species	Infested Cattle (*n* = 240)	Total Ticks (*n* = 3600)
*Rhipicephalus microplus*	204 (85%, 79.8–89.3) *	2530 (70.3%, 68.7–71.7)
*Rhipicephalus haemaphysaloides*	91 (37.9%, 31.7–44.4)	677 (18.8%, 17.5–20.1)
*Haemaphysalis bispinosa*	72 (30%, 24.3–36.2)	295 (8.2%, 7.3–9.1)
*Haemaphysalis spinigera*	28 (11.7%, 7.9–16.4)	90 (2.5%, 2.0–3.0)
*Amblyomma testudinarium*	7 (2.9%, 1.2–5.9)	7 (0.19%, 0.07–0.4)
*Ixodes* sp.	1	1

* Numbers in bracket represents percentage and 95% confidence interval, and *n* represents the total number.

**Table 4:** Tick infestation prevalence in cattle in Trashigang and Pema Gatshel districts.

Tick Species	Trashigang (*n* = 130)	Pema Gatshel (*n* = 110)	Infested Cattle (*n* = 240)	*χ^2^*	*p*-Value
*Rhipicephalus microplus*	96 (73.8%, 65.4–81.1) *	108 (98.2%, 93.6–99.8)	204	25.8	<0.001
*Rhipicephalus haemaphysaloides*	74 (56.9%, 47.9–65.6)	17 (15.4%, 9.3–23.6)	91	41.78	<0.001
*Haemaphysalis bispinosa*	39 (30%, 22.3–38.6)	33 (30%, 21.6–39.5)	72	0	1
*Haemaphysalis spinigera*	11 (8.5%, 4.3–14.6)	17 (15.4%, 9.3–23.6)	28	2.19	0.139

* Numbers in bracket represents percentage and 95% confidence interval.

**Table 5:** Characteristics of the explanatory variables (*n* = 240) used for the logistic regression analyses.

Variables	Categories	Trashigang (*n* = 130)	Pema Gatshel (*n* = 110)	Total (*n* = 240)	%	95%CI
Cattle age *	Adult	103	79	182	75.8	69.9–81.1
	Young	27	31	58	24.2	18.9–30.1
Cattle sex	Female	111	85	196	81.7	76.2–86.4
	Male	19	25	44	18.3	13.6–23.8
Cattle breed	European	73	80	153	63.8	57.3–69.8
	Indigenous	57	30	87	36.2	30.2–42.7
Altitude ^Ψ^	Mean	1687	1177			
	Range	897–2187	720–2060			
Latitude ^ϑ^	Mean	27.28	26.99			
	Range	27.11–27.41	26.84–27.12			
Longitude ^ϑ^	Mean	91.61	91.33			
	Range	91.44–91.76	91.09–91.46			

* The cattle under calf and heifer were categorized as “young” while the rest were categorized as adults (3 years and above), ^Ψ^ altitude in masl, ^ϑ^ latitude and longitude in decimal degrees. CI: confidence interval.

**Table 6:** The final multiple logistic regression models to understand factors associated with infestation prevalence of each tick species (categorized as infested or not) in cattle.

Variable	Estimate ± SE	OR (95% CI)	*p*-Value
*Rhipicephalus microplus*			
Intercept	506.59 ± 133.11		<0.001
Age (Young) ^ϖ^	1.72 ± 0.75	5.57 (1.5–35.5)	
Longitude × 10	−0.55 ± 0.15	0.57 (0.42–0.75)	
*Rhipicephalus haemaphysaloides*			
Intercept	−191.81 ± 27.31		0.003
Latitude × 10	0.70 ± 0.10	2.02 (1.67–2.48)	
*Haemaphysalis bispinosa*			
Intercept	−2.12 ± 0.55		0.009
Breed (Indigenous) ^μ^	0.62 ± 0.29	1.85 (1.04–3.29)	
Altitude/100	0.07 ± 0.04	1.07 (1.002–1.14)	
*Haemaphysalis spinigera*			
Intercept	0.35 ± 0.74		
Breed (Indigenous)	1.00 ± 0.43	2.72 (1.18–6.38)	<0.001
Altitude/100	−0.21 ± 0.06	0.81 (0.72–0.90)	

^ϖ^ Adult as the referent category, and ^μ^ European breed as the referent category. SE: standard error, OR: odds ratio, CI: confidence interval.

**Table 7:** Variable contribution, permutation importance, corrected Akaike information criteria (AICc), and number of parameters of the best MaxEnt models developed for predicting tick species occurrence in Eastern Bhutan.

Model	Variables	% Contribution	Permutation Importance	AICc	Parameters
*Rhipicephalus microplus*	DEM (Elevation)	56.1	76.4	3084.35	15
	LULC (Land use and land cover)	43.9	23.6		
*Rhipicephalus haemaphysaloides*	LULC (Land use and land cover)	46	9.4	1360.47	13
	Bio 16 (Precipitation of wettest quarter)	43.1	29.6		
	Bio 10 (Mean temperature of warmest quarter)	10.9	61		
*Haemaphysalis bispinosa*	LULC (Land use and land cover)	63.2	39.3	1095.32	13
	Bio 18 (Precipitation of warmest quarter)	30.6	44.7		
	Bio 16 (Precipitation of wettest quarter)	6.2	16.1		
*Haemaphysalis spinigera*	Bio 16 (Precipitation of wettest quarter)	45.7	26.3	433.91	8
	Bio 19 (Precipitation of coldest quarter)	33.4	66.9		
	LULC (Land use and land cover)	20.8	6.8		

## Data Availability

The data presented in this study are available in supplementary material here.

## Supplementary Materials

The following are available online at https://www.mdpi.com/2414-6366/6/1/27/s1: Figure S1. Response curve plotting the probability of *R. microplus* occurrence in Eastern Bhutan against the values of the top environmental variables: (A) elevation (DEM_SRTM) and (B) land use and land cover 2016 (LULC). The X-axis represents the variable value, and the Y-axis represents the probability of presence as predicted by the best MaxEnt model RM_B1. Figure S2. Response curves plotting the probability of *R. haemaphysaloides* occurrence in Eastern Bhutan against the values of the top environmental variables: (A) temperature of the warmest quarter (Bio 10), (B) land use and land cover 2016 (LULC), and (C) precipitation of the wettest quarter (Bio 16). The X-axis represents the variable value, and the Y-axis represents the probability of presence as predicted by the best MaxEnt model RH_D1. Figure S3. Response curves plotting the probability of *H. bispinosa* occurrence in Eastern Bhutan against the values of the top environmental variables: (A) precipitation of the warmest quarter (Bio 18), (B) land use and land cover 2016 (LULC), and (C) precipitation of the wettest quarter (Bio 16). The X-axis represents the variable value, and the Y-axis represents the probability of presence as predicted by the best MaxEnt model HB_C2. Figure S4. Response curves plotting the probability of *H. spinigera* occurrence in Eastern Bhutan against the values of the top environmental variables: (A) precipitation of the coldest quarter (Bio 19), (B) land use and land cover 2016 (LULC), and (C) precipitation of the wettest quarter (Bio 16). The X-axis represents the variable value, and the Y-axis represents the probability of presence as predicted by the best MaxEnt model HS_E1. Table S1. Results of simple logistic regression describing the relationships between infestation (infested or not) and cattle and geographic parameters. Table S2. Variable contribution and permutation importance values obtained during the first MaxEnt run performed with all the variables for predicting tick species presence. Only variables that achieved values of more than 1% in both metrics are shown. Table S3. Correlation matrix showing Spearman correlation coefficient (r_s_) for variables that achieved more than 1% contribution and permutation importance in the first MaxEnt run for all tick species. Table S4. MaxEnt models developed for predicting tick species occurrence in Eastern Bhutan. Table shows variables included into each different model. Variables, percent contribution (%), permutation importance (PI), AUC (training and testing), correlation (training and testing), corrected AIC, delta (Δ), Akaike weights (ω), and number of parameters (P) for each different model are given. The best model for each tick species is highlighted in bold. Table S5. Study area (in km^2^ and percentage) classified by the probability of tick species occurrence in Eastern Bhutan: RM_B1 for *R. microplus*; RH_D1 for *R. haemaphysaloides*; HB_C2 for *H. bispinosa*; and HS_E1 for *H. spinigera.* Dataset S1. Data (excel file) collected during the survey and used for analysis.
