# Supplementary material for: Identification, Distribution, and Habitat Suitability Models of Ixodid Tick Species in Cattle in Eastern Bhutan
Source: Trop Med Infect Dis. 2021 Feb 19;6(1):27. doi: 10.3390/tropicalmed6010027 (PMC7931079; doi:10.3390/tropicalmed6010027)
Supplement: Supplementary file 1 [file tropicalmed-06-00027-s001.zip › Supplementary files/Supplementary file_JN.docx]

**Supplementary materials**

**Figure S1.** Response curve plotting the probability of *R. microplus* occurrence in eastern Bhutan against the values of the top environmental variables: (A) Elevation (DEM_SRTM) and (B) Land use and land cover 2016 (LULC). The X-axis represents the variable value, and the Y-axis represents the probability of presence as predicted by the best MaxEnt model RM_B1.

**
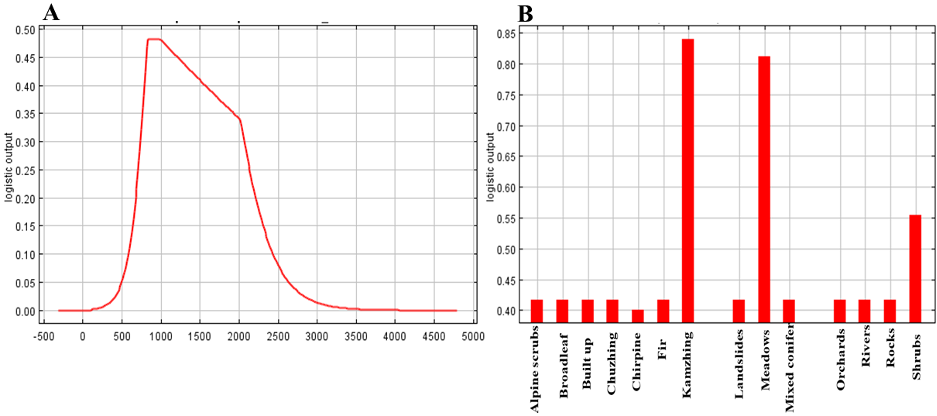
**

**Figure S2.** Response curves plotting the probability of *R. haemaphysaloides* occurrence in eastern Bhutan against the values of the top environmental variables: (A) Temperature of the warmest quarter (Bio 10), (B) Land use and land cover 2016 (LULC), and (C) precipitation of the wettest quarter (Bio 16). The X-axis represents the variable value, and the Y-axis represents the probability of presence as predicted by the best MaxEnt model RH_D1.

*
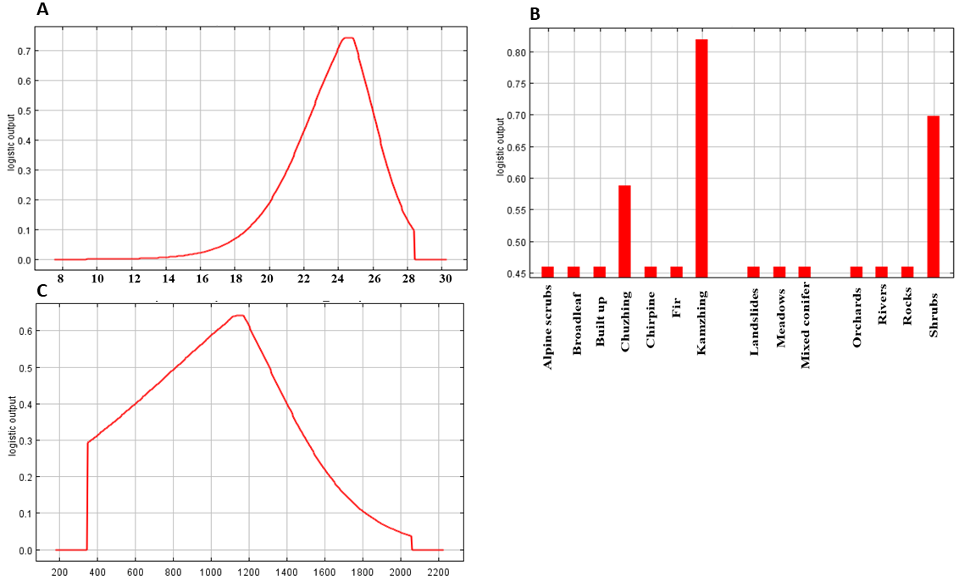
*

**Figure S3.** Response curves plotting the probability of *H. bispinosa* occurrence in eastern Bhutan against the values of the top environmental variables: (A) precipitation of the warmest quarter (Bio 18), (B) Land use and land cover 2016 (LULC), and (C) precipitation of the wettest quarter (Bio 16). The X-axis represents the variable value, and the Y-axis represents the probability of presence as predicted by the best MaxEnt model HB_C2.


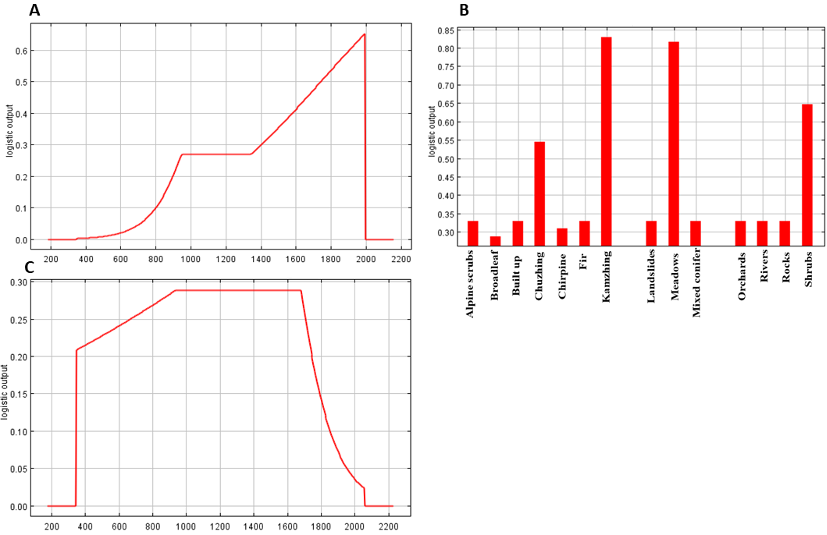


**Figure S4.** Response curves plotting the probability of *H. spinigera* occurrence in eastern Bhutan against the values of the top environmental variables: (A) precipitation of the coldest quarter (Bio 19), (B) Land use and land cover 2016 (LULC), and (C) precipitation of the wettest quarter (Bio 16). The X-axis represents the variable value, and the Y-axis represents the probability of presence as predicted by the best MaxEnt model HS_E1.

**
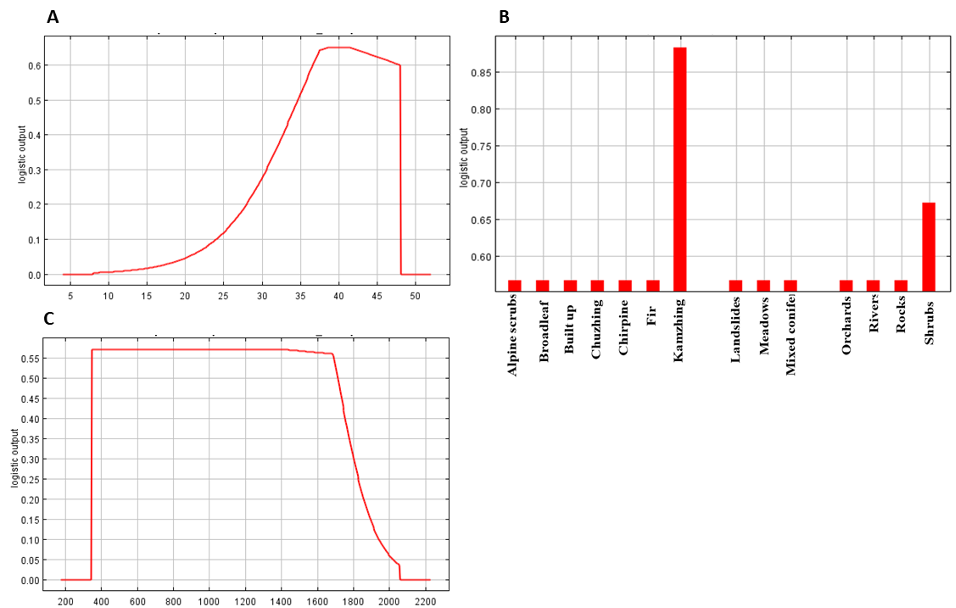
**

**Table S1.** Results of simple logistic regression describing the relationships between infestation (infested or not) and cattle and geographic parameters.

**Variable Intercept ± SE Slope ± SE χ^2^ P (>χ^2^) AIC**

***R. microplus* (n = 240)**

Age (Young) 1.4709 ± 0.1902 1.8614 ± 0.7443 10.2 0.001 196.7

Sex (Male) 1.6341 ± 0.1933 0.6685 ± 0.5588 1.6 0.232 205.3

Breed (Indigenous) 1.8383 ± 0.2349 -0.2697 ± 0.3684 0.5 0.464 206.4

Altitude 2.9909 ± 0.7240 -0.0836 ± 0.0452 3.5 0.064 203.4

Latitude 286.0783 ± 52.7624 -1.0440 ± 0.1932 53.6 <0.0001 153.3

Longitude 534.7555 ± 132.7182 -0.5823 ± 0.1449 20.4 <0.0001 186.5

***R. haemaphysaloides* (n = 240)**

Age (Young) -0.2877 ± 0.1498 -0.9540 ± 0.3487 8.3 0.004 314.3

Sex (Male) -0.4353 ± 0.1463 -0.3268 ± 0.3552 0.9 0.36 321.7

Breed (Indigenous) -0.6639 ± 0.1707 0.4562 ± 0.2750 2.8 0.09 319.8

Altitude -1.8970 ± 0.5159 0.0953 ± 0.0334 8.5 0.004 314.1

Latitude -191.8104 ± 27.3052 0.7042 ± 0.1004 63.1 <0.0001 259.5

Longitude -491.9548 ± 91.1948 0.5371 ± 0.0996 35.1 <0.0001 287.5

***H. bispinosa* (n = 240)**

Age (Young) -0.8630 ± 0.1623 0.0645 ± 0.33 0.0 0.84 297.2

Sex (Male) -0.7944 ± 0.1543 -0.3042 ± 0.38 0.7 0.42 296.6

Breed (Indigenous) -1.1073 ± 0.1871 0.6634 ± 0.29 5.3 0.02 292.0

Altitude -1.9666 ± 0.5439 0.0757 ± 0.03 4.8 0.03 292.4

Latitude -14.8673 ± 22.4159 0.0516 ± 0.08 0.4 0.53 296.8

Longitude -101.9797 ± 80.3645 0.1105 ± 0.09 1.6 0.20 295.6

***H. spinigera* (n = 240)**

Age (Young) -1.9334 ± 0.2231 -0.4274 ± 0.52 0.7 0.41 176.2

Sex (Male) -1.8777 ± 0.2106 -1.1668 ± 0.75 3.2 0.12 173.7

Breed (Indigenous) -2.3767 ± 0.2899 0.8081 ± 0.41 4.0 0.05 173.0

Altitude 0.6114 ± 0.7245 -0.1983 ± 0.06 14.2 0.0002 162.7

Latitude 7.6291 ± 31.9696 -0.0356 ± 0.12 0.1 0.76 176.8

Longitude 29.1316 ± 111.8207 -0.0341 ± 0.12 0.1 0.78 176.8

Altitude = meters altitude/100; Latitude = degrees latitude*10; Longitude = degrees longitude*10

**Table S2.** Variable contribution and permutation importance values obtained during the first MaxEnt run performed with all the variables for predicting tick species presence. Only variables that achieved values of more than 1% in both metrics are shown.

| **Variable** | **% contribution** | **Permutation importance** |
| --- | --- | --- |
| ***R. microplus*** |  |  |
| LULC | 44.1 | 28.5 |
| Bio 18 | 42.6 | 24.5 |
| DEM_SRTM | 9.6 | 28.2 |
| ***R. haemaphysaloides*** |  |  |
| LULC | 47.8 | 8 |
| Bio 18 | 31.6 | 24.5 |
| Bio 16 | 7.2 | 31.1 |
| Bio 10 | 2.1 | 28.9 |
| DEM_SRTM | 2 | 1.7 |
| Bio 8 | 1.7 | 25.1 |
| ***H. bispinosa*** |  |  |
| LULC | 63.9 | 39.9 |
| Bio 18 | 25.1 | 24 |
| Bio 12 | 4.2 | 9 |
| Bio 11 | 3.9 | 3.1 |
| Bio 16 | 1.2 | 9.4 |
| ***H. spinigera*** |  |  |
| Bio 19 | 29.7 | 55.7 |
| LULC | 22.4 | 11.7 |
| Bio 12 | 10.1 | 6.6 |
| Bio 17 | 3.5 | 2.3 |
| Bio 8 | 2.8 | 5.2 |
| Bio 11 | 2.5 | 5 |
| Bio 16 | 2.2 | 13.2 |

**Table S3.** Correlation matrix showing Spearman correlation coefficient (r_s_) for variables that achieved more than 1% contribution and permutation importance in the first MaxEnt run for all tick species.

| **Variables** |  |  |  |  |  |
| --- | --- | --- | --- | --- | --- |
| ***R. microplus*** |  |  |  |  | DEM_SRTM |
| Bio 18 |  |  |  |  | -0.93 |
| ***R. haemaphysaloides*** |  | Bio 16 | Bio 10 | Bio 8 | DEM_SRTM |
| Bio 18 |  | 1 | 0.91 | 0.91 | -0.94 |
| Bio 16 |  |  | 0.92 | 0.91 | -0.94 |
| Bio 10 |  |  |  | 1 | -0.99 |
| Bio 8 |  |  |  |  | -0.98 |
| ***H. bispinosa*** |  |  | Bio 12 | Bio 11 | Bio 16 |
| Bio 18 |  |  | 0.98 | 0.96 | 0.98 |
| Bio 12 |  |  |  | 0.97 | 1 |
| Bio 11 |  |  |  |  | 0.96 |
| ***H. spinigera*** | Bio 12 | Bio 17 | Bio 8 | Bio 11 | Bio 16 |
| Bio 19 | 0.85 | 1 | 0.97 | 0.97 | 0.84 |
| Bio 12 |  | 0.85 | 0.78 | 0.90 | 1 |
| Bio 17 |  |  | 0.97 | 0.97 | 0.84 |
| Bio 8 |  |  |  | 0.96 | 0.77 |
| Bio 11 |  |  |  |  | 0.89 |

**Table S4.** MaxEnt models developed for predicting tick species occurrence in eastern Bhutan. Table shows variables included into each different model. Variables, percent contribution (%), permutation importance (PI), AUC (training and testing), correlation (training and testing), corrected AIC, delta (Δ), Akaike weights (ω) and number of parameters (P) for each different model are given. The best model for each tick species is highlighted in bold.

| **Model** | **variable** | **%** | **PI** | **Train AUC** | **Train Cor** | **Test AUC** | **Test Cor** | **AICc** | **Δ** | **ω** | **P** |
| --- | --- | --- | --- | --- | --- | --- | --- | --- | --- | --- | --- |
| ***R. microplus*** | |  |  |  |  |  |  |  |  |  |  |
| RM_A | Bio 18 | 44.6 | 22 | 0.804 | 0.5 | 0.809 | 0.39 | 3093.02 | 8.67 | 0.01 | 19 |
|  | LULC | 42.8 | 32.1 |  |  |  |  |  |  |  |  |
|  | DEM | 12.6 | 45.9 |  |  |  |  |  |  |  |  |
| **RM_B1** | **DEM** | **56.1** | **76.4** | **0.803** | **0.5** | **0.809** | **0.39** | **3084.35** | **0** | **0.99** | **15** |
|  | **LULC** | **43.9** | **23.6** |  |  |  |  |  |  |  |  |
| RM_B2 | LULC | 50.9 | 34.6 | 0.774 | 0.46 | 0.795 | 0.4 | 3140.95 | 56.6 | 0 | 17 |
|  | Bio 18 | 49.1 | 65.4 |  |  |  |  |  |  |  |  |
| RM_B3 | DEM | 55.7 | 88.5 | 0.782 | 0.335 | 0.765 | 0.177 | 3535.91 | 451.6 | 0 | 20 |
|  | Bio 18 | 44.3 | 11.5 |  |  |  |  |  |  |  |  |
| ***R.haemaphysaloides*** | | |  |  |  |  |  |  |  |  |  |
| RH_A | LULC | 46.1 | 8.4 | 0.842 | 0.498 | 0.889 | 0.368 | 1371.98 | 11.59 | 0 | 17 |
|  | Bio 18 | 33.9 | 4.7 |  |  |  |  |  |  |  |  |
|  | Bio 16 | 9.7 | 24.7 |  |  |  |  |  |  |  |  |
|  | Bio 10 | 8.9 | 62.1 |  |  |  |  |  |  |  |  |
|  | Bio 8 | 1.4 | 0 |  |  |  |  |  |  |  |  |
|  | DEM | 0 | 0 |  |  |  |  |  |  |  |  |
| RH_B1 | LULC | 46.1 | 9.8 | 0.842 | 0.498 | 0.889 | 0.368 | 1372.13 | 11.74 | 0 | 17 |
|  | Bio 18 | 33.6 | 2.9 |  |  |  |  |  |  |  |  |
|  | Bio 16 | 9.7 | 34.7 |  |  |  |  |  |  |  |  |
|  | Bio 10 | 9.2 | 48.3 |  |  |  |  |  |  |  |  |
|  | Bio 8 | 1.4 | 4.3 |  |  |  |  |  |  |  |  |
| RH_B2 | LULC | 46.2 | 8.2 | 0.843 | 0.498 | 0.888 | 0.368 | 1363.19 | 2.8 | 0.76 | 14 |
|  | Bio 18 | 33.8 | 5.2 |  |  |  |  |  |  |  |  |
|  | Bio 10 | 10.8 | 62 |  |  |  |  |  |  |  |  |
|  | Bio 16 | 9.2 | 24.5 |  |  |  |  |  |  |  |  |
|  | DEM | 0 | 0 |  |  |  |  |  |  |  |  |
| RH_B3 | LULC | 46.1 | 7 | 0.842 | 0.497 | 0.888 | 0.368 | 1367.18 | 6.79 | 0.01 | 15 |
|  | Bio 18 | 33.5 | 4.9 |  |  |  |  |  |  |  |  |
|  | Bio16 | 10 | 29.4 |  |  |  |  |  |  |  |  |
|  | DEM | 6.6 | 4.9 |  |  |  |  |  |  |  |  |
|  | Bio 8 | 3.8 | 53.7 |  |  |  |  |  |  |  |  |
| RH_B4 | LULC | 47.8 | 15 | 0.846 | 0.5 | 0.887 | 0.363 | 1389.43 | 29.04 | 0 | 21 |
|  | Bio 18 | 36.1 | 23.4 |  |  |  |  |  |  |  |  |
|  | DEM | 11.2 | 5.7 |  |  |  |  |  |  |  |  |
|  | Bio 10 | 3.9 | 55.9 |  |  |  |  |  |  |  |  |
|  | Bio 8 | 1 | 0 |  |  |  |  |  |  |  |  |
| RH_B5 | LULC | 46.1 | 10.4 | 0.842 | 0.498 | 0.889 | 0.369 | 1368.93 | 8.54 | 0 | 16 |
|  | Bio 16 | 43.6 | 32.5 |  |  |  |  |  |  |  |  |
|  | Bio 10 | 8.9 | 56 |  |  |  |  |  |  |  |  |
|  | Bio 8 | 1.4 | 0.6 |  |  |  |  |  |  |  |  |
|  | DEM | 0 | 0 |  |  |  |  |  |  |  |  |
| RH_B6 | Bio 10 | 44.1 | 63.3 | 0.85 | 0.339 | 0.908 | 0.225 | 1573.14 | 212.8 | 0 | 24 |
|  | Bio 18 | 29.7 | 0 |  |  |  |  |  |  |  |  |
|  | Bio 16 | 17.3 | 34 |  |  |  |  |  |  |  |  |
|  | Bio 8 | 7.2 | 0.1 |  |  |  |  |  |  |  |  |
|  | DEM | 1.6 | 2.5 |  |  |  |  |  |  |  |  |
| RH_C1 | LULC | 46 | 9.4 | 0.842 | 0.498 | 0.888 | 0.368 | 1360.53 | 0.14 | 0.29 | 13 |
|  | Bio 18 | 33.8 | 5.7 |  |  |  |  |  |  |  |  |
|  | Bio 10 | 10.9 | 54.5 |  |  |  |  |  |  |  |  |
|  | Bio 16 | 9.3 | 30.4 |  |  |  |  |  |  |  |  |
| RH_C2 | LULC | 47.9 | 12.1 | 0.846 | 0.5 | 0.887 | 0.363 | 1382.92 | 22.53 | 0 | 19 |
|  | Bio 18 | 36.4 | 9.2 |  |  |  |  |  |  |  |  |
|  | DEM_SRTM | 11.1 | 9.9 |  |  |  |  |  |  |  |  |
|  | Bio 10 | 4.6 | 68.8 |  |  |  |  |  |  |  |  |
| RH_C3 | LULC | 47.8 | 13.2 | 0.837 | 0.482 | 0.886 | 0.35 | 1379.3 | 18.91 | 0 | 17 |
|  | Bio 18 | 34.7 | 2.1 |  |  |  |  |  |  |  |  |
|  | Bio 16 | 9 | 27.9 |  |  |  |  |  |  |  |  |
|  | DEM | 8.5 | 56.9 |  |  |  |  |  |  |  |  |
| RH_C4 | LULC | 46.2 | 8.4 | 0.843 | 0.498 | 0.888 | 0.369 | 1360.39 | 0 | 0.31 | 13 |
|  | Bio 16 | 43 | 32.9 |  |  |  |  |  |  |  |  |
|  | Bio 10 | 10.8 | 58.7 |  |  |  |  |  |  |  |  |
|  | DEM | 0 | 0 |  |  |  |  |  |  |  |  |
| RH_C5 | Bio 10 | 52.9 | 66.2 | 0.85 | 0.339 | 0.907 | 0.225 | 1559.08 | 198.7 | 0 | 20 |
|  | Bio 18 | 29.5 | 1.5 |  |  |  |  |  |  |  |  |
|  | Bio 16 | 16 | 29.2 |  |  |  |  |  |  |  |  |
|  | DEM | 1.7 | 3.1 |  |  |  |  |  |  |  |  |
| **RH_D1** | **LULC** | **46** | **9.4** | **0.842** | **0.498** | **0.889** | **0.368** | **1360.47** | **0.08** | **0.3** | **13** |
|  | **Bio 16** | **43.1** | **29.6** |  |  |  |  |  |  |  |  |
|  | **Bio 10** | **10.9** | **61** |  |  |  |  |  |  |  |  |
| RH_D2 | LULC | 47.8 | 11.9 | 0.837 | 0.482 | 0.886 | 0.35 | 1376.27 | 15.88 | 0 | 16 |
|  | Bio 16 | 43.7 | 31.4 |  |  |  |  |  |  |  |  |
|  | DEM | 8.5 | 56.8 |  |  |  |  |  |  |  |  |
| RH_D3 | Bio 10 | 53.6 | 61.4 | 0.85 | 0.339 | 0.907 | 0.225 | 1549.62 | 189.2 | 0 | 17 |
|  | Bio16 | 45.6 | 38.1 |  |  |  |  |  |  |  |  |
|  | DEM | 0.8 | 0.5 |  |  |  |  |  |  |  |  |
| RH_E1 | LULC | 52.8 | 25 | 0.804 | 0.47 | 0.819 | 0.314 | 1369.86 | 9.47 | 0 | 9 |
|  | Bio 10 | 47.2 | 75 |  |  |  |  |  |  |  |  |
| RH_E2 | Bio 16 | 50.6 | 67.1 | 0.817 | 0.465 | 0.857 | 0.329 | 1376.04 | 15.65 | 0 | 10 |
|  | LULC | 49.4 | 32.9 |  |  |  |  |  |  |  |  |
| RH_E3 | Bio 10 | 54.5 | 69.6 | 0.849 | 0.339 | 0.908 | 0.226 | 1546.92 | 186.5 | 0 | 16 |
|  | Bio 16 | 45.5 | 30.4 |  |  |  |  |  |  |  |  |
| ***H. bispinosa*** | |  |  |  |  |  |  |  |  |  |  |
| HB_A | LULC | 62.5 | 42.2 | 0.858 | 0.499 | 0.89 | 0.329 | 1120.82 | 25.5 | 0 | 21 |
|  | Bio 18 | 28.7 | 38 |  |  |  |  |  |  |  |  |
|  | Bio 11 | 7.3 | 8.5 |  |  |  |  |  |  |  |  |
|  | Bio 16 | 1.2 | 5.8 |  |  |  |  |  |  |  |  |
|  | Bio 12 | 0.3 | 5.5 |  |  |  |  |  |  |  |  |
| HB_B1 | LULC | 62.5 | 44.7 | 0.858 | 0.498 | 0.891 | 0.328 | 1113.35 | 18.03 | 0 | 19 |
|  | Bio 18 | 29 | 26.9 |  |  |  |  |  |  |  |  |
|  | Bio 11 | 7.4 | 12 |  |  |  |  |  |  |  |  |
|  | Bio 16 | 1.2 | 16.3 |  |  |  |  |  |  |  |  |
| HB_B2 | LULC | 62.8 | 32.9 | 0.859 | 0.497 | 0.889 | 0.326 | 1117.68 | 22.36 | 0 | 20 |
|  | Bio 18 | 28.9 | 40.8 |  |  |  |  |  |  |  |  |
|  | Bio 11 | 7.4 | 22.5 |  |  |  |  |  |  |  |  |
|  | Bio 12 | 1 | 3.8 |  |  |  |  |  |  |  |  |
| HB_B3 | LULC | 63.6 | 27.6 | 0.855 | 0.497 | 0.895 | 0.329 | 1098.69 | 3.37 | 0.15 | 14 |
|  | Bio 18 | 30.7 | 55.3 |  |  |  |  |  |  |  |  |
|  | Bio 12 | 3.7 | 0 |  |  |  |  |  |  |  |  |
|  | Bio 16 | 1.9 | 17.1 |  |  |  |  |  |  |  |  |
| HB_B4 | LULC | 62.4 | 31.9 | 0.863 | 0.504 | 0.891 | 0.332 | 1108.65 | 13.33 | 0 |  |
|  | Bio 16 | 28.5 | 40.8 |  |  |  |  |  |  |  |  |
|  | Bio 11 | 7.3 | 14.7 |  |  |  |  |  |  |  |  |
|  | Bio 12 | 1.8 | 12.6 |  |  |  |  |  |  |  |  |
| HB_B5 | Bio 11 | 50.6 | 59.4 | 0.817 | 0.226 | 0.834 | 0.121 | 1296.89 | 201.6 | 0 | 21 |
|  | Bio 12 | 45.1 | 17.6 |  |  |  |  |  |  |  |  |
|  | Bio 16 | 3.7 | 20.3 |  |  |  |  |  |  |  |  |
|  | Bio 18 | 0.5 | 2.8 |  |  |  |  |  |  |  |  |
| HB_C1 | LULC | 64 | 34.6 | 0.849 | 0.489 | 0.891 | 0.326 | 1103.79 | 8.47 | 0.01 | 15 |
|  | Bio 18 | 30.7 | 51.3 |  |  |  |  |  |  |  |  |
|  | Bio 12 | 5.3 | 14.1 |  |  |  |  |  |  |  |  |
| **HB_C2** | **LULC** | **63.2** | **39.3** | **0.857** | **0.497** | **0.895** | **0.329** | **1095.32** | **0** | **0.83** | **13** |
|  | **Bio 18** | **30.6** | **44.7** |  |  |  |  |  |  |  |  |
|  | **Bio 16** | **6.2** | **16.1** |  |  |  |  |  |  |  |  |
| HB_C3 | LULC | 64.5 | 29.2 | 0.846 | 0.498 | 0.866 | 0.332 | 1119.18 | 23.86 | 0 | 19 |
|  | Bio 16 | 29.3 | 54.8 |  |  |  |  |  |  |  |  |
|  | Bio 12 | 6.1 | 16 |  |  |  |  |  |  |  |  |
| HB_C4 | Bio 12 | 63.9 | 39.6 | 0.772 | 0.19 | 0.764 | 0.099 | 1318.68 | 223.4 | 0 | 20 |
|  | Bio 16 | 25.7 | 34.4 |  |  |  |  |  |  |  |  |
|  | Bio 18 | 10.4 | 26 |  |  |  |  |  |  |  |  |
| HB_D1 | LULC | 64.2 | 40.5 | 0.845 | 0.497 | 0.866 | 0.327 | 1108.85 | 13.53 | 0 | 16 |
|  | Bio 16 | 35.8 | 59.5 |  |  |  |  |  |  |  |  |
| HB_D2 | LULC | 68.8 | 45.2 | 0.846 | 0.491 | 0.88 | 0.328 | 1105.49 | 10.17 | 0.01 | 13 |
|  | Bio 18 | 31.4 | 54.8 |  |  |  |  |  |  |  |  |
| HB_D3 | Bio 18 | 73 | 64.7 | 0.772 | 0.189 | 0.758 | 0.098 | 1327.17 | 231.9 | 0 | 22 |
|  | Bio 16 | 27 | 35.3 |  |  |  |  |  |  |  |  |
| ***H. spinigera*** | |  |  |  |  |  |  |  |  |  |  |
| HS_A | Bio 19 | 33 | 53.2 | 0.884 | 0.371 | 0.853 | 0.191 | 446.19 | 13.02 | 0 | 11 |
|  | Bio16 | 29 | 0.4 |  |  |  |  |  |  |  |  |
|  | LULC | 19.4 | 12.2 |  |  |  |  |  |  |  |  |
|  | Bio 12 | 11.7 | 21.3 |  |  |  |  |  |  |  |  |
|  | Bio 11 | 3.5 | 4.9 |  |  |  |  |  |  |  |  |
|  | Bio 8 | 3 | 5.9 |  |  |  |  |  |  |  |  |
|  | Bio 17 | 0.3 | 2.2 |  |  |  |  |  |  |  |  |
| HS_B1 | Bio 19 | 33.4 | 54.3 | 0.884 | 0.371 | 0.853 | 0.192 | 440.62 | 7.45 | 0.01 | 10 |
|  | Bio 16 | 29 | 5.3 |  |  |  |  |  |  |  |  |
|  | LULC | 19.4 | 7.3 |  |  |  |  |  |  |  |  |
|  | Bio 12 | 11.7 | 18.1 |  |  |  |  |  |  |  |  |
|  | Bio 11 | 3.5 | 10.4 |  |  |  |  |  |  |  |  |
|  | Bio 8 | 3 | 4.6 |  |  |  |  |  |  |  |  |
| HS_B2 | Bio 19 | 34.9 | 40.2 | 0.878 | 0.355 | 0.858 | 0.186 | 447.64 | 14.47 | 0 | 11 |
|  | Bio 16 | 29.5 | 16.5 |  |  |  |  |  |  |  |  |
|  | LULC | 20.3 | 13.6 |  |  |  |  |  |  |  |  |
|  | Bio 12 | 11.7 | 2.5 |  |  |  |  |  |  |  |  |
|  | Bio 11 | 2.9 | 6.2 |  |  |  |  |  |  |  |  |
|  | Bio 17 | 0.7 | 21 |  |  |  |  |  |  |  |  |
| HS_B3 | Bio 19 | 33.8 | 57.3 | 0.875 | 0.359 | 0.855 | 0.19 | 442.55 | 9.38 | 0 | 10 |
|  | Bio 16 | 29.7 | 1.5 |  |  |  |  |  |  |  |  |
|  | LULC | 19.9 | 11.8 |  |  |  |  |  |  |  |  |
|  | Bio 12 | 14.1 | 27 |  |  |  |  |  |  |  |  |
|  | Bio 8 | 1.8 | 1.2 |  |  |  |  |  |  |  |  |
|  | Bio 17 | 0.8 | 1.2 |  |  |  |  |  |  |  |  |
| HS_B4 | Bio 16 | 40.3 | 27 | 0.885 | 0.372 | 0.852 | 0.193 | 446.2 | 13.03 |  | 11 |
|  | Bio 19 | 28.3 | 61.7 |  |  |  |  |  |  |  |  |
|  | LULC | 19.8 | 7.9 |  |  |  |  |  |  |  |  |
|  | Bio 11 | 5 | 1 |  |  |  |  |  |  |  |  |
|  | Bio 17 | 3.7 | 2.4 |  |  |  |  |  |  |  |  |
|  | Bio 8 | 2.9 | 0 |  |  |  |  |  |  |  |  |
| HS_B5 | Bio 12 | 40.4 | 20.8 | 0.884 | 0.369 | 0.852 | 0.188 | 446.65 | 13.48 | 0 | 11 |
|  | Bio 19 | 33.3 | 58.5 |  |  |  |  |  |  |  |  |
|  | LULC | 19.5 | 5.8 |  |  |  |  |  |  |  |  |
|  | Bio 11 | 3.5 | 7.9 |  |  |  |  |  |  |  |  |
|  | Bio 8 | 3 | 4.1 |  |  |  |  |  |  |  |  |
|  | Bio 17 | 0.3 | 2.8 |  |  |  |  |  |  |  |  |
| HS_B6 | Bio 17 | 33.4 | 64.2 | 0.884 | 0.371 | 0.851 | 0.192 | 440.63 | 7.46 | 0.01 | 10 |
|  | Bio16 | 29 | 3.7 |  |  |  |  |  |  |  |  |
|  | LULC | 19.4 | 5.9 |  |  |  |  |  |  |  |  |
|  | Bio 12 | 11.7 | 19.6 |  |  |  |  |  |  |  |  |
|  | Bio 11 | 3.5 | 6.6 |  |  |  |  |  |  |  |  |
|  | Bio 8 | 3 | 0 |  |  |  |  |  |  |  |  |
| HS_B7 | Bio 19 | 42.6 | 15.1 | 0.866 | 0.202 | 0.861 | 0.107 | 507.87 | 74.7 | 0 | 11 |
|  | Bio 11 | 32.8 | 59.4 |  |  |  |  |  |  |  |  |
|  | Bio 12 | 17.3 | 0 |  |  |  |  |  |  |  |  |
|  | Bio 17 | 3.1 | 0 |  |  |  |  |  |  |  |  |
|  | Bio 16 | 2.1 | 10.6 |  |  |  |  |  |  |  |  |
|  | Bio 8 | 2 | 14.9 |  |  |  |  |  |  |  |  |
| HS_C1 | Bio 19 | 35.7 | 83.5 | 0.878 | 0.355 | 0.858 | 0.185 | 437.16 | 3.99 | 0.03 | 9 |
|  | Bio 16 | 29.7 | 5.1 |  |  |  |  |  |  |  |  |
|  | LULC | 20.1 | 6.7 |  |  |  |  |  |  |  |  |
|  | Bio 12 | 11.7 | 4 |  |  |  |  |  |  |  |  |
|  | Bio 11 | 2.9 | 0.7 |  |  |  |  |  |  |  |  |
| HS_C2 | Bio 19 | 34.4 | 69.4 | 0.875 | 0.359 | 0.855 | 0.19 | 433.17 | 0 | 0.21 | 8 |
|  | Bio 16 | 29.7 | 3.7 |  |  |  |  |  |  |  |  |
|  | LULC | 19.8 | 6.5 |  |  |  |  |  |  |  |  |
|  | Bio 12 | 14.1 | 20 |  |  |  |  |  |  |  |  |
|  | Bio 8 | 2 | 0.4 |  |  |  |  |  |  |  |  |
| HS_C3 | Bio 16 | 41.7 | 24.5 | 0.885 | 0.372 | 0.852 | 0.193 | 435.73 | 2.56 | 0.06 | 9 |
|  | Bio 19 | 31.1 | 59.7 |  |  |  |  |  |  |  |  |
|  | LULC | 19.5 | 10.1 |  |  |  |  |  |  |  |  |
|  | Bio 11 | 4.6 | 2.1 |  |  |  |  |  |  |  |  |
|  | Bio 8 | 3.1 | 3.6 |  |  |  |  |  |  |  |  |
| HS_C4 | Bio 12 | 40.4 | 24.8 | 0.884 | 0.369 | 0.852 | 0.188 | 441.09 | 7.92 | 0 | 10 |
|  | Bio 19 | 33.6 | 63.4 |  |  |  |  |  |  |  |  |
|  | LULC | 19.5 | 5.7 |  |  |  |  |  |  |  |  |
|  | Bio 11 | 3.5 | 5.5 |  |  |  |  |  |  |  |  |
|  | Bio 8 | 3 | 0.6 |  |  |  |  |  |  |  |  |
| HS_C5 | Bio 16 | 49.5 | 11.3 | 0.881 | 0.366 | 0.844 | 0.179 | 479.26 | 46.09 | 0 | 15 |
|  | LULC | 19.2 | 8.3 |  |  |  |  |  |  |  |  |
|  | Bio 12 | 14.7 | 14.2 |  |  |  |  |  |  |  |  |
|  | Bio 8 | 11.4 | 59.9 |  |  |  |  |  |  |  |  |
|  | Bio 11 | 5.2 | 6.3 |  |  |  |  |  |  |  |  |
| HS_C6 | Bio 19 | 45.8 | 28.6 | 0.867 | 0.201 | 0.862 | 0.107 | 502.29 | 69.12 | 0 | 10 |
|  | Bio 11 | 32.8 | 40.1 |  |  |  |  |  |  |  |  |
|  | Bio 12 | 17.3 | 0 |  |  |  |  |  |  |  |  |
|  | Bio 16 | 2.1 | 10.1 |  |  |  |  |  |  |  |  |
|  | Bio 8 | 2 | 21.2 |  |  |  |  |  |  |  |  |
| HS_D1 | Bio 19 | 35.9 | 89.4 | 0.873 | 0.349 | 0.859 | 0.186 | 434.02 | 0.85 | 0.14 | 8 |
|  | Bio 16 | 22.9 | 0 |  |  |  |  |  |  |  |  |
|  | LULC | 20.2 | 6.4 |  |  |  |  |  |  |  |  |
|  | Bio 12 | 14 | 4.2 |  |  |  |  |  |  |  |  |
| HS_D2 | Bio 16 | 46.2 | 22.1 | 0.877 | 0.361 | 0.857 | 0.193 | 433.24 | 0.07 | 0.2 | 8 |
|  | Bio 19 | 32 | 68.5 |  |  |  |  |  |  |  |  |
|  | LULC | 19.9 | 8.2 |  |  |  |  |  |  |  |  |
|  | Bio 8 | 2 | 1.2 |  |  |  |  |  |  |  |  |
| HS_D3 | Bio 12 | 43.5 | 27 | 0.875 | 0.357 | 0.853 | 0.186 | 437.99 | 4.82 | 0.02 | 9 |
|  | Bio 19 | 34.5 | 60.3 |  |  |  |  |  |  |  |  |
|  | LULC | 19.9 | 11.3 |  |  |  |  |  |  |  |  |
|  | Bio 8 | 2 | 1.3 |  |  |  |  |  |  |  |  |
| HS_D4 | Bio 16 | 52.3 | 9.1 | 0.867 | 0.349 | 0.844 | 0.181 | 482.03 | 48.86 | 0 | 15 |
|  | LULC | 20 | 7.9 |  |  |  |  |  |  |  |  |
|  | Bio 12 | 18.6 | 24.8 |  |  |  |  |  |  |  |  |
|  | Bio 8 | 9.1 | 58.2 |  |  |  |  |  |  |  |  |
| HS_D5 | Bio 19 | 45.2 | 52.7 | 0.835 | 0.179 | 0.865 | 0.103 | 497.88 | 64.71 | 0 | 8 |
|  | Bio 12 | 27.3 | 0 |  |  |  |  |  |  |  |  |
|  | Bio 8 | 27.2 | 27.3 |  |  |  |  |  |  |  |  |
|  | Bio 16 | 0.4 | 20.1 |  |  |  |  |  |  |  |  |
| **HS_E1** | **Bio 16** | **45.7** | **26.3** | **0.871** | **0.352** | **0.861** | **0.188** | **433.91** | **0.74** | **0.15** | **8** |
|  | **Bio 19** | **33.4** | **66.9** |  |  |  |  |  |  |  |  |
|  | **LULC** | **20.8** | **6.8** |  |  |  |  |  |  |  |  |
| HS_E2 | Bio 16 | 71.2 | 16.6 | 0.866 | 0.35 | 0.847 | 0.187 | 493.63 | 60.46 | 0 | 16 |
|  | LULC | 21 | 6 |  |  |  |  |  |  |  |  |
|  | Bio 8 | 7.7 | 77.3 |  |  |  |  |  |  |  |  |
| HS_E3 | Bio 19 | 74.7 | 87.4 | 0.861 | 0.325 | 0.817 | 0.149 | 435.62 | 2.45 | 0.06 | 7 |
|  | LULC | 25.3 | 12.6 |  |  |  |  |  |  |  |  |
|  | Bio 8 | 0 | 0 |  |  |  |  |  |  |  |  |
| HS_E4 | Bio 19 | 48.6 | 43.5 | 0.835 | 0.179 | 0.865 | 0.103 | 507.23 | 74.06 | 0 | 10 |
|  | Bio 8 | 27.1 | 40 |  |  |  |  |  |  |  |  |
|  | Bio 16 | 24.3 | 16.4 |  |  |  |  |  |  |  |  |
| HS_F1 | Bio 16 | 76.7 | 93.5 | 0.855 | 0.314 | 0.859 | 0.162 | 436.04 | 2.87 | 0.05 | 7 |
|  | LULC | 23.3 | 6.5 |  |  |  |  |  |  |  |  |
| HS_F2 | Bio 19 | 74.4 | 83.4 | 0.859 | 0.325 | 0.811 | 0.15 | 435.53 | 2.36 | 0.06 | 7 |
|  | LULC | 25.6 | 16.6 |  |  |  |  |  |  |  |  |
| HS_F3 | Bio 19 | 78.1 | 88.7 | 0.821 | 0.168 | 0.858 | 0.099 | 489.08 | 55.91 | 0 | 5 |
|  | Bio 16 | 21.9 | 11.3 |  |  |  |  |  |  |  |  |

**Table S5.** Study area (in km^2^ and percentage) classified by the probability of tick species occurrence in eastern Bhutan: RM_B1 for *R. microplus*; RH_D1 for *R. haemaphysaloides*; HB_C2 for *H. bispinosa*; and HS_E1 for *H. spinigera.*

|  |  |  |  | **Probability class** | |  |  |  |  |  |
| --- | --- | --- | --- | --- | --- | --- | --- | --- | --- | --- |
| **Model** | **1 (0-0.2)** |  | **2 (0.2-0.4)** | | **3 (0.4-0.6)** |  | **4 (0.6-0.8)** | | **5 (0.8-1)** | |
|  | **km^2^** | **%** | **km^2^** | **%** | **km^2^** | **%** | **km^2^** | **%** | **km^2^** | **%** |
| RM_B1 | 1028.5 | 40.4 | 251.9 | 9.9 | 1028.8 | 40.4 | 166.9 | 6.5 | 70 | 2.7 |
| RH_D1 | 1119.9 | 43.9 | 728.2 | 28.6 | 467.8 | 18.4 | 134.4 | 5.3 | 95.9 | 3.8 |
| HB_C2 | 955.4 | 37.5 | 957 | 37.6 | 502.2 | 19.7 | 19.8 | 0.8 | 111.7 | 4.4 |
| HS_E1 | 1252.8 | 49.2 | 448.5 | 17.6 | 423.5 | 16.6 | 268.2 | 10.5 | 152.9 | 6 |
